# The Forgotten First Contact: The Oral Cavity as a Site of Local Biotransformation Shaping the Chemical Form of Polyphenolic Drugs and Dietary Compounds

**DOI:** 10.3390/ph19101661

**Published:** 2026-10-21

**Authors:** Caterina Nela Dumitru, Teodora Marcu, Alina Oana Dumitru, Mădălina Nicoleta Matei

**Affiliations:** 1 Research Centre in the Medical-Pharmaceutical Field, Faculty of Medicine and Pharmacy, “Dunărea de Jos” University of Galați, 35 Al. I. Cuza Street, 800010 Galați, Romania; caterina.dumitru@ugal.ro (C.N.D.); madalina.matei@ugal.ro (M.N.M.); 2 Faculty of Medicine and Pharmacy, “Dunărea de Jos” University of Galați, 35 Al. I. Cuza Street, 800010 Galați, Romania

**Keywords:** local biotransformation, polyphenols, microbial biotransformation, oral microbiome, oral metabotype, free fraction, topical oral administration, phytochemical–drug interaction

## Abstract

**Background/Objectives:** Polyphenols are dietary constituents and active substances in dosage forms applied locally in the mouth, yet their bioavailability is interpreted almost exclusively in terms of intestinal absorption and colonic microbial metabolism. This review examines the oral cavity as a site of local biotransformation, a compartment that can chemically modify polyphenols before absorption, and asks whether this conversion could be measured as a phenotype. **Methods:** Narrative synthesis is reported according to SANRA. Four databases were searched from inception to 31 August 2026. **Results:** Flavonoid glucosides are hydrolysed to aglycones within minutes of contact with saliva; anthocyanins are deglycosylated to cyanidin and converted to protocatechuic acid; epigallocatechin gallate is de-esterified. Local concentrations reach 2–145 μM, two orders of magnitude above plasma. Conversion capacity varied more than twenty-fold among 17 subjects in the only quantitative study. **Conclusions:** The oral cavity can alter the chemical form of the polyphenols presented to absorption. The orally transformed fraction of an ingested dose is small; a first-pass effect is not demonstrated, and the established consequences are local and qualitative. Oral metabotypes have not been demonstrated; the concept and its operational classification are proposed as hypotheses to be tested in adequately sized cohorts.

## 1. Introduction

Polyphenolic compounds are relevant to pharmacy both as food constituents and as active substances in medicinal products. They are the largest class of phytochemicals in the human diet, with an estimated daily intake of 0.5 to 1 g in European populations [1], and they are, at the same time, active principles in dosage forms applied locally in the oral cavity via mouthwashes, gels, dental varnishes and mucoadhesive systems intended for the supragingival biofilm and the periodontal pocket. Biomedical interest in them rests on a substantial body of preclinical evidence for antioxidant, anti-inflammatory, antimicrobial and chemopreventive activity [1].

This interest has, however, consistently met a well-documented pharmacokinetic obstacle. The systemic bioavailability of most polyphenols is low—plasma concentrations of the parent compounds rarely exceeding the nanomolar or submicromolar range, that is, one to three orders of magnitude below the concentrations at which effects are demonstrated in vitro [1,2]. This gap, often referred to as the polyphenol bioavailability paradox, has defined the research agenda of the field for the past two decades [2,3].

The dominant solution proposed for this paradox has been the gut microbiota. The unabsorbed fraction reaches the colon, where the resident bacterial community catabolises it into low-molecular-weight metabolites, often better absorbed and more biologically active than their precursors [2]. The production of these metabolites varies widely among individuals, generating distinct metabotypes that determine responsiveness to dietary intervention [3]. Urolithin metabotypes, derived from ellagitannins through the action of taxa such as *Gordonibacter* spp., have become the canonical model of this relationship [4,5]. The same logic—microbiota as a determinant of the effective exposure to an active substance—has recently been extended to non-antibiotic drugs, under the name of pharmacomicrobiomics [6].

This conceptual construction contains, however, an anatomical omission. The route of a polyphenol does not begin in the colon but in the oral cavity. The oral microbiome is the second most complex microbial community of the human body, with over 600 prevalent species-level taxa organised into structured biofilms on dental, mucosal and lingual surfaces [7]. The hydrolytic activities of the type found in the colon, glycosidase and esterase activities, have been documented in human saliva [8,9,10], and tannases have been characterised in oral bacteria (Section 2.2.3), whereas the decarboxylases and reductases that complete colonic catabolism have not been tested in the oral cavity. Nevertheless, pharmacokinetic models treat the oral cavity as a mere transit conduit [1,2].

The correct formulation of the problem is not that the phenomenon has gone unnoticed. The individual pieces of evidence have existed for two decades, but they are scattered across four bodies of literature with non-convergent terminologies that do not cite one another: nutritional pharmacokinetics, sensory science, oral cancer chemoprevention and oral microbiology. What is missing is a common consequence, which the present work formulates.

Evidence that the oral cavity is more than a transit conduit has been available for two decades. In a landmark study, incubation of human saliva with quercetin 4’-glucoside or genistein 7-glucoside produced the corresponding aglycone within minutes. Glucose conjugates were rapidly hydrolysed, whereas rutin, quercitrin and naringin remained stable, and interindividual variability in the hydrolysis of genistein 7-glucoside exceeded twenty-fold across the seventeen subjects tested [8]. The following observation was subsequently extended: eugenyl-β-D-glucoside is completely degraded on incubation with human saliva, and the addition of antibiotics stops the degradation, confirming the microbiological origin of the hydrolysis [9].

The relevance of this biotransformation is amplified by the local concentration profile. After consumption of green tea equivalent to two or three cups, peak salivary concentrations of epigallocatechin (11.7 to 43.9 μg/mL), epigallocatechin gallate (4.8 to 22 μg/mL) and epicatechin (1.8 to 7.5 μg/mL) were reached within minutes and exceeded plasma levels by two orders of magnitude, with a salivary half-life of 10 to 20 min [10]. Salivary catechins were not detectable when the same tea solids were given in capsules [10], so oral exposure depends on the dosage form (Section 2.1.3). A second set of human data confirmed the profile: holding green tea leaves or black tea infusion in the mouth generated salivary catechin concentrations between 2.2 and 131.0 μM, with significant interindividual variation in peak levels but a similar kinetic profile across subjects [11].

At these concentrations, in the range of a few up to approximately 145 μM, the thresholds at which polyphenols exert their reported effects in vitro are met [1]. To our knowledge, the oral cavity is the only human compartment in which concentrations in this range have been measured directly, by serial sampling [10,11], whereas colonic luminal concentrations are largely estimated [2].

The interaction is, however, bidirectional, and polyphenols are also modulators of the community that transforms them (Section 2.5). To this is added a further layer of biopharmaceutical complexity: epigallocatechin gallate precipitates salivary proteins, including α-amylase, S100 and cystatins, with relatively high affinity (Kd = 2.74 × 10^−6^ M) [12]. Sequestration by protein binding limits the free fraction available both for antimicrobial action and for metabolism, a classical biopharmaceutical problem, which, to our knowledge, has not been addressed systematically for saliva.

By analogy with the intestinal metabotypes [3,5], the hydrolytic variability described above raises the possibility of oral metabotypes, that is, discrete metabolic phenotypes defined by conversion capacity. This possibility has not been tested. No study has measured the population distribution of oral conversion capacity, and its candidate determinants, biofilm composition, periodontal status, salivary flow, oral hygiene and antiseptic use, are inferred from indirect evidence [8,9]. If the hypothesis were confirmed, it could explain part of the heterogeneity of results in intervention studies [3] and would identify a modifiable determinant of response.

A terminological distinction is required at the outset, because the three phenomena discussed here are frequently conflated. We use local oral biotransformation for chemical conversion demonstrated within the oral cavity itself, an event whose primary relevance is to local mucosal and biofilm exposure. We use presystemic modification of chemical species for the observation that the compound presented to absorption may no longer be the compound ingested, a qualitative change that is independent of the mass fraction involved. We reserve first-pass effect for a presystemic conversion or loss large enough to alter systemic pharmacokinetic parameters, in the conventional sense applied to intestinal and hepatic metabolism. The evidence reviewed here supports the first two categories; it does not establish the third, and the quantitative arguments bearing on it are set out in the section titled The Quantitative Limit of the Oral Contribution. These three levels are kept distinct throughout the text, figures and tables.

Two further terms are used in a fixed sense. Oral hydrolytic capacity (OHC) denotes a measurable quantity, the rate at which an individual’s saliva converts a standard glycosidic probe substrate (Section 2.4.5). Oral metabotype denotes the hypothesis that OHC values fall into discrete categories; it is used only where this hypothesis is meant, and never as a synonym for oral biotransformation or for OHC.

The aim of this work is to examine the oral cavity as a site of local biotransformation of polyphenolic compounds, to assess whether the interindividual variability of this conversion could be treated as a measurable phenotype, and to outline, graded by the strength of the evidence, what would follow from this for locally administered polyphenolic preparations and for their interactions with medication.

The individual observations summarised in Section 2.1, Section 2.2 and Section 2.3, salivary deglycosylation of flavonoid glucosides, de-esterification of epigallocatechin gallate, the oral handling of anthocyanins and the interindividual variability of these conversions, are published findings [8,9,10,11] and are presented here as a synthesis. Three elements are proposals of the authors and are identified as such wherever they appear: the description of the oral cavity in biopharmaceutical terms, through contact time, free fraction and surface retention; the oral metabotype, with an operational classification and predictions by which it can be refuted (Section 2.4); and a bidirectional model in which locally formed metabolites act back on the community that produced them (Section 2.5). The proposed oral metabotype also differs operationally from the classifications with which it is most often compared. Intestinal microbial metabotypes are assigned from the presence of terminal metabolites in urine or faeces collected over one or more days, and pharmacogenetic metaboliser classes are fixed by the host genotype; the oral classification is assigned from the rate of a single hydrolytic step measured in saliva within 30 min and is expected to change within days (Section 2.4.3). The pharmaceutical implications in Section 2.6 are graded by the strength of the supporting evidence.

## 2. Results

### 2.1. The Oral Compartment as a Biopharmaceutical System

#### 2.1.1. Microbial Niches and Biofilm Organisation

The oral cavity is not a single habitat but an assembly of distinct micro-habitats, comprising the teeth, gingival sulcus, tongue, cheeks, hard and soft palate and tonsils, colonised by bacterial communities in which distinct subsets predominate in each habitat [7]. Analysis of 36,043 16S rRNA clones identified 1179 taxa, of which 24% were named, 8% cultivated but unnamed and 68% uncultivated phylotypes; 259, 413 and 875 taxa, respectively, are required to account for 90%, 95% and 99% of the clones examined [7]. Updated databases raise the number of oral taxa to approximately 770 [13].

This structuring has direct metabolic consequences. Unlike the colonic lumen, where the substrate encounters a relatively homogenised community, in the oral cavity a polyphenol dissolved in saliva successively traverses zones with different enzymatic profiles: the supragingival biofilm dominated by *Streptococcus* and *Actinomyces*, the tongue dorsum, which is the niche with the greatest mucosal biomass and the most intense reductive activity [14], and the gingival sulcus, anaerobic and dominated by Bacteroidetes and Fusobacteria. Bacterial densities are on the order of 10^8^–10^9^ cells/mL in whole saliva and 10^11^ cells/g in mature dental plaque [15]. The spatial organisation of these consortia has been mapped at the micrometre scale, and the biofilm structure with its own pH and oxygen gradients is well characterised [16,17]. Biotransformation, therefore, does not occur in the oral cavity as a unitary entity but in parallel, in compartments with different enzymatic capacities, and the final product measured in saliva is a resultant.

#### 2.1.2. Saliva as a Reaction Medium and the Free-Fraction Problem

Unstimulated salivary flow in healthy adults is 0.3–0.4 mL/min, rising to 1–2 mL/min under masticatory or gustatory stimulation, with a total volume of 0.5–1.5 L/24 h [18]. The pH of whole saliva lies between 6.2 and 7.6, with buffering capacity provided predominantly by the bicarbonate system in stimulated saliva and by phosphate and proteins in unstimulated saliva.

The protein composition of saliva is not neutral towards polyphenols, and the consequence is eminently biopharmaceutical. The mucins MUC5B, gel-forming, and MUC7, non-gel-forming, constitute the protective barrier of the mucosa [19]; the mean MUC5B concentration in saliva is approximately 200 μg/mL but may be up to five-fold higher in the mucosal pellicle lining the soft tissues [20]. Epigallocatechin gallate substantially alters the properties of both the MUC5B polymer network and monomeric MUC7, and at MUC5B concentrations of 200–1600 μg/mL it produces a significant increase in mucin viscosity [20].

To this is added binding to enzymes and defence proteins. Epigallocatechin gallate precipitates several salivary proteins, including α-amylase, S100 and cystatins, binds α-amylase with Kd = 2.74 × 10^−6^ M and inhibits it non-competitively, limiting the formation of fermentable substrate; at the same time, α-amylase reduces its antimicrobial activity against *Aggregatibacter actinomycetemcomitans* [12].

The polyphenol–salivary protein interaction is a general phenomenon, extensively characterised in astringency research: proline-rich proteins, histatins and amylase form complexes and precipitates with tannins and with epigallocatechin gallate, and affinity depends on the degree of galloylation and polymerisation [21,22]. The consequence for any assessment of local exposure is direct and, in our view, systematically underestimated in the existing literature. The salivary matrix simultaneously exerts a sequestering effect, which reduces the free fraction, and a target-modulating effect. The free fraction, and not the total concentration measured by extraction, is the pharmacologically relevant quantity. Given the order of magnitude of the Kd, at physiological salivary concentrations of mucins and amylase the bound fraction is non-negligible, and reporting total concentration systematically overestimates effective exposure. This distinction is standard in plasma pharmacokinetics and absent from salivary pharmacokinetics.

A third level of interaction is the redox-enzymatic one, through salivary peroxidase and myeloperoxidase derived from leukocytes migrating into the gingival sulcus, whose activity is increased by exposure to polyphenols [23]. Since this level has been treated separately and in detail [24], we retain it here only as a mechanism by which polyphenols alter the selective pressure on the community through the host.

#### 2.1.3. Compartment Kinetics

The temporal profile of oral exposure differs radically from the systemic one: a high, short peak, with C_max_ in the 2–145 μM range, t_1/2_ of 10–20 min and repeated exposure three to six times daily, depending on the dietary pattern [10,11]. The local area under the curve is substantial despite the short duration.

Decoupling from systemic exposure has been demonstrated directly and has formulation significance: holding the solution in the mouth without swallowing generated higher salivary concentrations, whereas administration of the same tea solids in capsules produced no detectable salivary levels [10]. Compartment exposure is, therefore, a function of the dosage form, not of the ingested dose.

**Figure 1:**
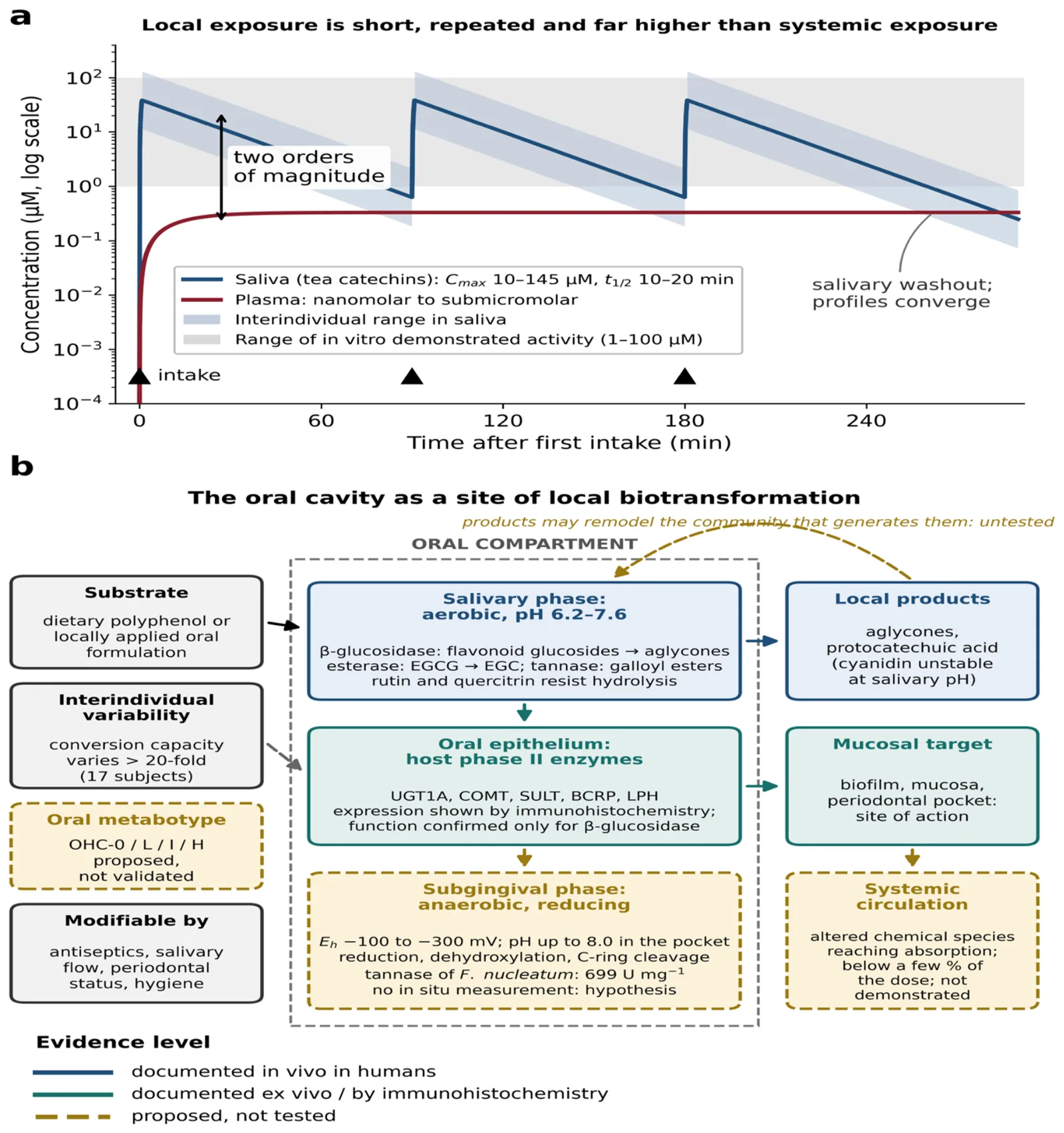
Local biotransformation of polyphenolic compounds in the oral cavity: demonstrated elements and proposed extensions: (**a**) concentration–time profiles after repeated intake (black triangles mark the intake times, at 0, 90 and 180 min). Salivary catechin concentrations peak within minutes at 2–145 μM (shaded band: interindividual range; solid line: representative profile) and decay with a half-life of 10–20 min, whereas plasma concentrations remain in the nanomolar to submicromolar range; the gray band marks the concentration range at which in vitro activity is demonstrated. Data from [10,11]. (**b**) Functional organisation of the compartment. The substrate encounters two spatially separated metabolic regimes, an aerobic hydrolytic salivary phase and an anaerobic reductive subgingival phase, superimposed on a host epithelium that expresses phase II enzymes and efflux transporters (demonstrated immunohistochemically; catalytic function in situ confirmed only for β-glucosidase). Conversion capacity varied more than twenty-fold among the 17 subjects of the only quantitative study and is presumed modifiable by antiseptics, salivary flow and periodontal status, which is the basis of the proposed oral metabotype. The dashed return arrow denotes the recursive step formulated here and not yet tested: the aglycones generated by the community are more potent modulators of that same community than their parent glycosides. Line colour and style encode the level of evidence. Solid black arrows indicate the direction of substrate flow and biotransformation; the gray dashed arrow indicates a modulating influence (interindividual variability) rather than a material flow. Elements identified as proposed, namely, the oral metabotype, the recursive step and the systemic consequences of antiseptic suppression, are hypotheses formulated here and are not established findings.

Clearance is governed by the Dawes model of salivary elimination, in which the residual salivary volume after swallowing, approximately 0.8 mL, and the salivary flow rate determine the exponential dilution rate of any solute [25]. The practical consequence is that a reduced salivary flow prolongs contact time and extends the window of microbial biotransformation, a clinical variable developed in Section 2.6.6. The resulting exposure profile, compared with the plasma profile on the same time axis, is shown in Figure 1a.

#### 2.1.4. Substantivity and the Acquired Pellicle

Retention outlasts the duration of presence in the liquid phase. Polyphenols adsorb onto the acquired pellicle and onto mucosal surfaces, forming a slow-release reservoir. Studies of oral catechin retention have quantified concentrations on the order of tens of nanograms per milligram of mucosa 60 min after ingestion [26], indicating biphasic kinetics with a rapid salivary phase and a slow tissue phase. The composition and functions of the acquired pellicle are well described, and the adsorption of tea polyphenols onto the salivary pellicle has been measured directly by ellipsometry [27,28,29]. The adsorbed fraction remains in prolonged contact with the biofilm and is probably the one relevant to biotransformation, although no study has directly measured the conversion rate in this phase. For mucoadhesive dosage forms, this is the phase that determines the effective duration of exposure at the site.

#### 2.1.5. Gingival Crevicular Fluid as a Second Compartment

The gingival sulcus and the periodontal pocket have distinct physicochemical properties. Gingival crevicular fluid is an exudate of serum origin, with a flow rate of approximately 0.05–0.1 μL/min per site in health, increasing 10- to 30-fold in inflammation [30]. The pH in periodontal pockets rises from approximately 6.9 to 7.4–8.0 in advanced periodontitis, and the redox potential falls from +200 or +300 mV in young supragingival plaque to −100 or −300 mV in mature subgingival biofilm [15,31].

These alkaline, strongly reducing and anaerobic conditions favour reductive reactions, dehydroxylations and hydrogenation of the C2–C3 double bond, analogous to the colonic ones, unlike the hydrolytic reactions that predominate in the aerobic salivary phase. The oral cavity, thus, contains, within the same organ, two complementary metabolic regimes. Gingival crevicular fluid also constitutes a route of entry for systemically absorbed polyphenols back into the oral cavity, closing a local–systemic–local circuit that has not been characterised to date.

#### 2.1.6. Comparative Synthesis

The parameters discussed so far can be placed alongside their colonic counterparts (Table 1). On the available data, the two compartments appear to differ in regime rather than only in degree: a residence time three orders of magnitude shorter, a redox potential of the opposite sign in the salivary phase, and hydrolytic reactions where the colon performs reductions. Cells marked as undetermined are not bibliographic omissions but genuine gaps in the literature, developed in Section 2.2.5 and Section 2.8.

### 2.2. The Enzymatic Repertoire of the Oral Compartment and Its Taxonomic Mapping

#### 2.2.1. Historical Note

The presence of glycosidase enzymes in human saliva was demonstrated as early as 1954 [36], and intraoral hydrolysis of glucosides was observed and correlated with taste perception in the late 1990s. Characterisation of β-glucosidase activity in species-level identified oral bacteria is, however, much more recent [37]. This chronology explains an anomaly of the field: the phenomenon was described for decades in the food science and sensory perception literature without being integrated into polyphenol pharmacokinetics. The two bodies of literature evolved in parallel, and the present work also seeks their reunification.

The enzymatic repertoire of the compartment comprises four functional classes: hydrolytic glycosidases, esterases and tannases, host phase II enzymes and transporters present in the oral epithelium, and oxidoreductases. The first three are directly relevant to the local biotransformation of the substrate.

#### 2.2.2. Glycosidases

Deglycosylation is the best characterised reaction. Incubation of human saliva with quercetin 4’-glucoside or genistein 7-glucoside produces the corresponding aglycones within minutes [8], and the substrate specificity is strict: glucose conjugates are rapidly hydrolysed, whereas rutin, quercitrin and naringin remain intact [8]. The origin of the activity is dual. The bacterial contribution is supported by the inhibition produced by antibacterials in vivo and in vitro and by experiments on subcultured oral colonies, but cytosol isolated from oral epithelial cells is itself capable of efficient hydrolysis [8]. The phenomenon, therefore, proceeds through two parallel mechanisms, whose relative contributions remain unquantified.

Extension to other matrices confirmed the generality of the reaction. Eugenyl-β-D-glucoside is completely degraded on incubation with human saliva, and the addition of antibiotics stops the degradation [9]. In the field of aroma precursors, the human oral microbiota hydrolyses glycosidic precursors from grapes, releasing odorant compounds, a capacity that is bacteria-dependent and shows different profiles among individuals [38]; the same capacity was subsequently demonstrated for glycosidic precursors from other fruit matrices [39].

Molecular characterisation has begun recently. Of seven candidate enzymes from oral bacteria produced recombinantly and screened for glycosidase activity, only the β-glucosidase from *Prevotella* sp. (PsBG1) showed activity, hydrolysing chromogenic substrates and aroma glucosides with release of salicylaldehyde, hexanol and octanol; the work includes the crystal structure, kinetic parameters and testing in artificial saliva [40]. The result is doubly significant: it confirms enzyme–taxon attribution and, at the same time, suggests that this capacity is less widespread in the oral community than is assumed.

#### 2.2.3. Esterases and Tannases

Hydrolysis of the galloyl ester bond is the second transformation documented in vivo. The study of salivary catechin concentrations revealed catechin esterase-like activity in saliva, converting epigallocatechin gallate to epigallocatechin; holding a solution of epigallocatechin gallate in the mouth generated both the parent compound and epigallocatechin in saliva and, subsequently, epigallocatechin in urine [10]. The reaction is pharmacokinetically relevant, because the two compounds differ significantly in protein affinity, permeability and antimicrobial activity profile. The responsible enzyme has not been identified, and its bacterial, epithelial or glandular origin remains unknown.

Tannin acyl hydrolases (tannases, EC 3.1.1.20) constitute the second relevant class of esterases, and here the literature offers an informative contrast. Two tannases have been identified and biochemically characterised in oral taxa. TanAAp, encoded by the apar_1020 gene of *Atopobium parvulum*, a species abundant on the tongue dorsum, shows optimal activity at pH 6.0 and a broad thermal range, both compatible with oral processing, but has the lowest specific activity among the described bacterial tannases (3.5 U/mg) and does not hydrolyse complex tannins such as tannic acid [41]. TanBFnp, from *Fusobacterium nucleatum* subsp. *polymorphum*, lies at the opposite extreme: at 699 U/mg on methyl gallate, it is one of the most active bacterial tannases known, 76.5% above the *Lactobacillus plantarum* reference enzyme, and it retains full activity after prolonged incubation up to 45 °C [42].

The contrast has direct ecological significance. The aerobic lingual commensal has marginal tannase capacity, whereas the subgingival anaerobe, abundant in mature plaque and in the periodontal pocket, possesses one of the strongest activities described. This independently supports the distinction between the two metabolic regimes of the same organ (Section 2.1.5 and Section 2.2.5) and provides a concrete molecular mechanism for the subgingival regime.

The gap remains, but it is more precise than an absence of data. Both enzymes were characterised as recombinant proteins, on model substrates. No measurement has been performed on whole saliva, on intact human biofilm or on dietary polyphenolic substrates, and the contribution of tannases to the de-esterification of epigallocatechin gallate, the reaction documented in vivo in humans [10], has never been assessed.

#### 2.2.4. Host Phase II Enzymes and Efflux Transporters

The most relevant body of evidence comes from oral cancer chemoprevention. Analysis of 15 human oral mucosal tissues revealed the presence of key metabolic enzymes, distributed within surface epithelia and terminal salivary ducts, and β-glucosidase assays confirmed that both whole saliva and microflora-reduced saliva deglycosylate anthocyanins [35]. The enzyme panel identified immunohistochemically comprises β-glucosidase, UDP-glucuronosyltransferases 1A, breast cancer resistance protein, sodium-dependent glucose co-transporter, lactase-phlorizin hydrolase and catechol-O-methyltransferase, localised predominantly in the basal and spinous layers with exclusion of the cornified layer; lactase-phlorizin hydrolase and catechol-O-methyltransferase are also present in the epithelium of the salivary ducts [35].

The role of these enzymes is established in the small intestine, where lactase-phlorizin hydrolase and cytosolic β-glucosidase carry out deglycosylation as an obligatory step in flavonoid absorption [43,44]. The authors concluded that the hydrolytic enzymes, phase II enzymes and efflux transporters required for local enteric recycling are present in human oral mucosa, comparable to the small intestine [35]. Enteric recycling is a term taken from intestinal pharmacology: a compound is taken up and conjugated by epithelial cells, the conjugate is effluxed back into the lumen, and it is deconjugated and taken up again. In the oral cavity, this cycle has not been demonstrated experimentally. Only its first steps have been observed: deglycosylation and the appearance of glucuronidated anthocyanins in saliva after rinsing [35]; the efflux and recycling steps are inferred from protein expression. That conclusion rests on immunohistochemical detection of the proteins together with functional confirmation of β-glucosidase activity; glucuronidation, methylation and BCRP-mediated efflux of polyphenols have not themselves been measured in human oral tissue. Saliva samples collected after rinsing contained glucuronidated anthocyanin conjugates, consistent with intracellular uptake and phase II conversion [35].

The oral cavity, therefore, possesses the molecular apparatus of a metabolic compartment and not only that of microbial biotransformation. We note the methodological limit, and it is retained wherever this evidence is invoked below: immunohistochemical identification establishes the presence and expression of the protein, not catalytic activity in situ; the only functionally confirmed activity in this set is the β-glucosidase activity. Statements elsewhere in this work concerning phase II capacity and local enteric recycling in the oral cavity are to be read in that sense, as inferred from expression rather than demonstrated by measurement of flux on polyphenolic substrates.

#### 2.2.5. The Subgingival Reductive Regime as an Unvalidated Hypothesis

The anaerobic, strongly reducing conditions described in Section 2.1.5 are, in theory, compatible with the dehydroxylation, C2–C3 double-bond hydrogenation and C-ring cleavage reactions characteristic of colonic catabolism. No study has, however, tested whether the subgingival biofilm performs these transformations. The hypothesis is testable: anaerobic incubation of fresh subgingival plaque with (+)-catechin or with ellagic acid, with LC-MS/MS detection of phenyl-γ-valerolactone- or urolithin-type products, would provide a direct answer. Phenyl-γ-valerolactones are the dominant colonic metabolites of flavan-3-ols and have analytical standards and validated methods available, which makes their detection immediately applicable [45]. A positive result would mean that a second human site produces urolithins, with implications for the entire metabotype paradigm.

#### 2.2.6. Taxonomic Mapping

The enzymes documented in the oral compartment are collected in Table 2, together with the substrate, product, origin and level of evidence for each. Nitrate reductase, which does not act on polyphenols, is included as a reference activity: it is the only oral microbial biotransformation for which the responsible taxa, the in vivo activity and a systemic consequence are all established (Section 2.6.1), and it provides the benchmark against which the polyphenol-converting activities are compared. The column on origin deserves to be read first, since it separates activities attributed to an identified taxon from those established only functionally, on whole saliva.

Enzyme–taxon attribution has begun but remains incomplete in a specific way. None of the existing attributions concerns dietary polyphenolic substrates, flavonoid glucosides, anthocyanins or galloylated catechins. None was obtained in intact biofilm or in whole saliva but exclusively on recombinant enzymes. In addition, decisively for the argument of the present work, none has been correlated with the metabolic phenotype of the donor. The functional evidence available on human saliva is aggregate, and the taxonomic evidence comes from heterologous systems; the two have never been brought together in the same subject. The situation contrasts with the intestinal literature, where species such as *Gordonibacter urolithinfaciens* have been identified as urolithin producers, with characterisation of the responsible enzymes and validation of the correlation with the individual metabotype [4,5]. This gap defines the first methodological priority of the field and is directly addressable: functional metagenomics applied to salivary samples, correlated with metabolic phenotyping by LC-MS/MS, followed by validation on isolates.

#### 2.2.7. An Unresolved Contradiction: The Case of Rutin

The literature contains a discrepancy with major practical consequences. The reference data indicate that rutin is resistant to salivary hydrolysis [8], whereas other sources state that salivary β-glucosidase activity hydrolyses flavonoid glycosides including rutin [35,49]. The discrepancy may reflect differences in incubation time, in donor microbiota composition or in analytical sensitivity.

A third explanatory factor, not considered to date, is enzyme-source specificity. If the hydrolysis of simple glucosides is performed predominantly by PsBG1-type β-glucosidases [40], and rutin requires an α-rhamnosidase to remove the rhamnosyl moiety before glucosidic hydrolysis, then the discrepancy would reflect the variable presence of a second enzymatic activity rather than protocol differences. The hypothesis is testable by parallel incubation of rutin and isoquercitrin in the same donors.

Resolution is not academic: rutin is the dominant flavonoid of several widely consumed functional foods, and its status as a substrate determines whether rutin intake is bioactivated before swallowing.

### 2.3. Polyphenolic Substrate Classes in the Oral Compartment

The six major classes are surveyed below on the same grid: the transformations are documented in the oral compartment and the specific gap. The contrast among classes is itself informative, because the degree of characterisation falls abruptly from flavonoid glycosides towards hydrolysable tannins, without this gradient reflecting biological importance.

#### 2.3.1. Flavonoid Glycosides

Flavonoid glycosides is the best-documented class and, at the same time, the one that demonstrates the structural selectivity of the compartment. Quercetin 4’-glucoside and genistein 7-glucoside are hydrolysed to aglycones within minutes, whereas rutin, quercitrin and naringin remain intact [8]. The determinant is the nature of the sugar moiety: glucose conjugates are substrates, those with rhamnose, rutinose or neohesperidose are not. The same rule governs intestinal absorption, where the nature of the sugar has proved to be the major determinant of the bioavailability of flavonoid glycosides in humans [50].

The consequence has, to our knowledge, not previously been formulated in exposure terms. European flavonoid intake is composed of fractions with divergent oral fates: one part is bioactivated locally, another traverses the compartment unmodified. The case of rutin is illustrative, being the dominant flavonoid of buckwheat (*Fagopyrum esculentum*) and its derived products, in which germination substantially alters both the total content and the qualitative profile of the phenolic fraction [51]. Current bioavailability models do not make this distinction.

The resulting aglycone is not an indifferent product: quercetin and genistein inhibit, in vitro, the proliferation of oral cancer cells [8], which places salivary deglycosylation on the trajectory of a local chemopreventive mechanism.

#### 2.3.2. Anthocyanins

The only class for which the oral compartment has been pharmacokinetically characterised in a clinical context is whole saliva and microflora-reduced saliva deglycosylate black raspberry anthocyanins [35]. Deglycosylation substantially alters the properties of the compound: removal of the bulky sugar moiety eliminates steric hindrance, introduces an additional hydroxyl group capable of radical scavenging and facilitates transporter-independent cellular uptake [35]. The resulting aglycone is not, however, a terminal product. At the pH of whole saliva, 6.2–7.6, the flavylium cation converts to the colourless chalcone form and undergoes C-ring cleavage, generating protocatechuic acid from the B-ring and phloroglucinaldehyde from the A-ring; in cell-free medium at physiological pH, approximately 96% of free cyanidin degrades within four hours, through a spontaneous, cell-independent process [52].

The oral compartment, therefore, produces a three-step sequence rather than a single reaction: glycoside, then ephemeral cyanidin, and then protocatechuic acid. Human data confirm the route. An LC-MS/MS analysis of saliva collected after rinsing revealed retention of the parent anthocyanins and of protocatechuic acid, described by the authors as a stable and functional metabolite, for up to four hours [35]. Protocatechuic acid is, therefore, likely to be the dominant measurable product of the oral compartment for this class, and bioactivation must be understood as the local generation of a stable, absorbable metabolite rather than as accumulation of the aglycone. Anthocyanin structure additionally determines both susceptibility to microbial degradation and bioavailability at the buccal mucosa, which links the substrate class directly to the local absorption route discussed in Section 2.6.5 [53].

This reinterpretation also explains part of the non-enzymatic degradation reported ex vivo for bilberry, chokeberry, black raspberry, red grape and strawberry extracts [49], which was attributed to structure and to oral bacteria without being separated from the intrinsic chemical instability at neutral pH. No study has correlated deglycosylation capacity with the sequencing-determined oral microbiota composition of the donor.

The phase II conjugation documented in Section 2.2.4 applies directly to this class. The authors’ original formulation deserves emphasis, since it anticipates the present thesis: interindividual differences in anthocyanin bioactivation and in enteric recycling capacity would influence treatment, because retention of bioactivated compounds at the target site would support therapeutic efficacy [35].

#### 2.3.3. Catechins and Galloyl Esters

The class with the best-characterised concentration profile (Section 2.1.3) and with two documented reactions: de-esterification of epigallocatechin gallate by catechin esterase-like activity and the downstream transformations of the non-esterified catechins [10]. Holding a solution of epigallocatechin gallate in the mouth generated the parent compound and epigallocatechin in saliva, and, subsequently, epigallocatechin in urine [10], a sequence consistent with entry of an orally formed metabolite into the systemic circulation and its subsequent excretion. The design does not, however, exclude de-esterification of the swallowed fraction in the gastrointestinal tract, and the magnitude of the oral contribution to the systemic metabolic profile, therefore, remains unquantified.

Catechins also illustrate the three competing fates of a polyphenol in the oral cavity: biotransformation by esterase activity; sequestration by binding to salivary proteins, since epigallocatechin gallate precipitates α-amylase, S100 and cystatins [12]; and retention on the acquired pellicle and on the mucosa [26], to which is added interaction with the mucin network [20]. These processes are concurrent, and the ratio among them determines the fraction effectively available for each. No study has measured them simultaneously, which constitutes, in our view, the most important biopharmaceutical gap in the field.

#### 2.3.4. Proanthocyanidins

Proanthocyanidins are a class with an information profile inverted relative to the preceding ones: the effects on the biofilm are well characterised, but the biotransformation is entirely unknown. Purified, chemically defined cranberry proanthocyanidin fractions reduced *Streptococcus mutans* biofilm formation in vitro and dental caries development in vivo in rats, an effect attributed to bioactive A-type dimers and oligomers; specific oligomers, from dimers to dodecamers, at 0.1 mg/mL, diminished insoluble glucan synthesis by surface-adsorbed GtfB on saliva-coated hydroxyapatite and impaired bacterial glycolysis [54]. Proanthocyanidins and flavonols, separately or in combination, inhibit surface-adsorbed glucosyltransferases and F-ATPases, as well as acid production [55]. Cranberry flavonoids, particularly A-type proanthocyanidins and myricetin, inhibit glucosyltransferase activity and exopolysaccharide-mediated adherence without killing the microorganisms [56].

This last formulation is essential to the bidirectional argument of the work: polyphenols can functionally restructure the biofilm without a bactericidal effect, that is, by altering its phenotype rather than its composition through selection. There are no data on the depolymerisation or catabolism of proanthocyanidins in the oral compartment. The contrast with the colon is stark: there, polymeric proanthocyanidins are catabolised by the human microbiota into low-molecular-weight phenolic acids, and the absorption and metabolism of the class have been synthesised [57,58]. The antiperiodontal effects of cranberry proanthocyanidins have also been confirmed by independent groups [59]. Oligomers with a high degree of polymerisation are unlikely to be absorbed, which makes the oral cavity and the biofilm their quasi-exclusive site of action, an observation with direct consequences for the formulation of cranberry-based preparations intended for oral use.

#### 2.3.5. Ellagitannins and Ellagic Acid

These have the most pronounced contrast in the field. The colonic metabolism of ellagitannins is the canonical paradigm of microbial biotransformation [4,5,60], and urolithins circulate in plasma as glucuronide and sulfate conjugates at concentrations of 0.2–20 μM [60,61]. In parallel, the relevance of urolithins to oral pathology has been demonstrated: urolithin A, a gut microbiota-derived metabolite, attenuates periodontitis in mice through dual anti-inflammatory and osteoclastogenesis-inhibiting action, in a ligature-induced model, with reductions in TNF-α and IL-6 and protection of the alveolar bone [62].

A paradoxical situation results. A metabolite produced by the gut microbiota is effective in an oral disease, and no one has checked whether the oral microbiota can produce it locally. The periodontal pocket offers precisely the anaerobic and reducing conditions required for the dehydroxylation reactions involved (Section 2.1.5 and Section 2.2.5), and the identification of a highly active tannase in an abundant subgingival anaerobe [42] adds biochemical plausibility to the first step of the pathway. There is no study of ellagitannins, ellagic acid or urolithins in the oral compartment. We consider this one of the most informative open questions: a positive answer would mean local formation of a potentially therapeutic metabolite at the pathological site, although the concentrations that could be reached there are unknown.

#### 2.3.6. Hydroxycinnamic Acids and Simple Phenolic Compounds

Chlorogenic acid, the ester of caffeic acid with quinic acid and the dominant phenolic of coffee, is an obvious esterase substrate with well-characterised human pharmacokinetics after coffee consumption [63,64], and its hydrolysis to caffeic acid should occur orally through the same type of activity documented for epigallocatechin gallate. Experimental confirmation is lacking. Similarly, the decarboxylation of phenolic acids, a bacterial reaction well characterised in other systems, has not been tested orally. In the colon, bacteria capable of hydrolysing chlorogenic acid have been isolated and characterised, and the enzymes carrying out the subsequent steps, phenolic acid decarboxylases, vinyl phenol reductases and hydroxycinnamate reductases, have been identified at the molecular level [65,66]. For the oral cavity, there is no equivalent. The only substrate in this area for which evidence exists is that of volatile phenolic glycosides: the human oral microbiota hydrolyses them, releasing terpenes, benzyl alcohol and C6 alcohols, a bacteria-dependent capacity with a profile that varies among individuals [38,39].

#### 2.3.7. Comparative Characterisation of the Six Polyphenol Classes

The six classes can be ordered by the degree of characterisation achieved in the oral compartment (Table 3). The gradient is steep and does not follow biological importance: the first three classes have in vivo human evidence, the last three have none.

The column on correlation with the donor microbiota is entirely negative. This summarises the state of the field: it is known that biotransformation occurs and, in part, who can perform it, but the link has never been established between the capacity of an individual’s community and the conversion that individual achieves. Section 2.4 turns this gap into a conceptual framework.

### 2.4. Oral Metabotypes: Proposal of a Conceptual Framework

#### 2.4.1. The Metabotype Paradigm

The term metabotype is a contraction of metabolic phenotype and denotes the classification of individuals into discrete categories according to their capacity to generate a specific metabolite from a given substrate. The discrete character of the categories is part of the definition, not an incidental property: a unimodal distribution of conversion capacity describes interindividual variability, not metabotypes. This requirement is what turns the concept into a falsifiable claim and underlies the first prediction formulated in Section 2.4.6.

The closest analogy in the pharmaceutical domain is the metaboliser phenotype of pharmacogenetics, where patients are classified as poor, intermediate, extensive or ultrarapid metabolisers for a cytochrome P450 isoform, with direct consequences for dosing. The classification logic is identical, and the difference concerns exclusively the determinant, which here is the microbial genome rather than that of the host. The distinction has, however, a major practical consequence: unlike the CYP genotype, which is fixed, the microbial composition is modifiable, all the more so the oral one, an argument developed in Section 2.4.3.

The observation that individuals exposed to the same polyphenol intake produce different metabolites has led, over the past decade, to a classification scheme based on conversion capacity rather than on exposure. Three systems have been validated in cohorts: equol producers and non-producers after isoflavone intake [67]; urolithin metabotypes derived from ellagitannins, with the canonical classification into producers of urolithin A only, producers of urolithin A, isourolithin A and urolithin B, and non-producers [5]; and, more recently, lunularin metabotypes derived from resveratrol, in a group of 195 healthy volunteers of whom 26% lacked the corresponding dehydroxylase activities [68]. What unites these systems is not the substrate but the logic: the phenotype is defined by what the individual produces, not by what he or she consumes [3].

The European Food Safety Authority has rejected most health claims relating to polyphenols, citing insufficient characterisation of the constituent, overly general wording of the claim and insufficient evidence to establish a cause-and-effect relationship; the claims for cocoa flavanols and for olive oil polyphenols constitute the authorised exceptions. Authors who have analysed this body of decisions have proposed interindividual variability of metabolism as the underlying explanation of the heterogeneity on which the negative assessments rested [69]. This interpretation assumes, however, implicitly, that the variability originates exclusively in the colon. This assumption has never been tested.

#### 2.4.2. Three Independent Lines of Evidence for Oral Variability

The existing literature contains, without ever having connected them, three independent demonstrations of interindividual variability in oral metabolic capacity. The first is quantified. In a limited group of 17 subjects, interindividual variability in the hydrolysis of genistein 7-glucoside exceeded twenty-fold [8]. The authors concluded that salivary hydrolysis of certain flavonoid glucosides may be important in some individuals and not in others, and that the high variability of this activity should be taken into account in future studies [8]. The recommendation, made in 2005, was not followed. A second set of human data confirmed significant interindividual variation in peak salivary concentrations of catechins and theaflavins, with a, nonetheless, similar kinetic profile [11], suggesting that variability affects the amplitude of exposure more than its shape.

The second is qualitative and bacterially attributed. The capacity of the human oral microbiota to hydrolyse glycosidic precursors is bacteria-dependent, and the odorant profile generated differs among individuals [38]. Variability here is not merely quantitative but qualitative: different individuals produce different sets of products from the same substrate, precisely the criterion that defines a metabotype, as opposed to a simple high- or low-producer gradient.

The third is clinical and has a therapeutic consequence. Data from an oral chemoprevention trial indicated that patient-specific differences in local retention and metabolism of black raspberry components affected therapeutic responsiveness [35].

The three observations come from disjoint fields, nutritional pharmacokinetics, sensory science and preventive oncology, and none has been interpreted as a manifestation of a single phenomenon. We propose this interpretation.

#### 2.4.3. Proposed Definition

We define the oral metabotype as the metabolic phenotype of an individual determined by the capacity of the oral microbial community and the oral epithelium to convert polyphenolic compounds into specific metabolites during buccal transit, independently of the intestinal metabolic capacity of the same individual. The definition is advanced as a hypothesis. No study has measured the population distribution of oral conversion capacity, and the available data do not permit the identification of discrete oral metabotypes; the term is, therefore, used throughout as the name of a proposed construct and not of a demonstrated phenotype.

Three elements distinguish the concept from its intestinal counterpart. The first is the dual component: unlike the intestinal metabotype, attributed exclusively to the microbiota, the oral one incorporates a documented epithelial contribution, through cytosolic hydrolysis [8] and phase II metabolism [35], making it a mixed host–microbiome phenotype. The second is the short temporal window: conversion occurs within minutes rather than hours, which means that the oral metabotype is expressed through the reaction rate rather than through the equilibrium yield, and that phenotyping requires kinetics rather than a single point measurement. The third is high modifiability: the oral microbiota is accessible to daily mechanical, antiseptic and professional intervention, unlike the colonic one. If the oral metabotype is clinically relevant, it is also correctable, which turns it from a descriptor into a therapeutic target.

Operationally, the proposed oral metabotype differs from the two classifications with which it is most often compared in what is measured, over what time and in which matrix. Intestinal microbial metabotypes are assigned from the presence and relative amounts of terminal products, urolithins, equol or lunularin, in urine or faeces collected over one or more days after a defined intake [5,67,68]; they describe the end point of a multistep catabolic pathway. Pharmacogenetic metaboliser classes are assigned from the host genotype, or from the metabolic ratio of a probe drug, and remain stable over the life of the individual. The oral classification proposed here would be assigned from the rate of a single hydrolytic step, measured in saliva within 30 min of contact with a standard probe substrate. It would reflect both microbial and epithelial enzymes and is expected to change within days after antiseptic use, antibiotic therapy or periodontal treatment. Table 4 extends this comparison.

#### 2.4.4. Candidate Determinants

The presumed determinants of the oral metabotype are listed, together with the proposed mechanism for each, in Table S1. They fall into two unequal categories. Periodontal status, salivary flow, oral hygiene and antiseptic use, smoking, plaque load, habitual diet and recent antibiotic therapy are all accessible to intervention. The only non-modifiable determinant identified is age and even this acts indirectly through the decline in salivary flow and the alteration of community composition. The predominance of modifiable determinants constitutes the translational argument of this work and sharply distinguishes the oral metabotype from its intestinal counterpart.

Four groups of factors are expected to modulate the extent of oral biotransformation, and each can be expressed through the parameters defined in Section 2.1. Their effects are inferred from the cited data and have not been measured in combination.

Composition. The structure of the substrate is the strongest determinant documented so far. The sugar moiety decides whether a flavonoid glycoside is hydrolysed at all: glucosides are converted within minutes, whereas rhamnosides, rutinosides and neohesperidosides are not [8]. Galloylation and the degree of polymerisation govern binding to salivary proteins and, therefore, the free fraction available for conversion [21,22]. The vehicle matters as well, since proteins, fibre and lipids in a food or formulation compete for the same binding interactions, and the mucin and amylase contents of saliva differ among individuals [19,20].

*Oral pH.* Salivary pH (6.2–7.6) and the more alkaline pH of the periodontal pocket [18,31] act at two levels. They determine chemical stability, which is critical for anthocyanins, whose flavylium cation is stable only at acidic pH and degrades spontaneously near neutrality [52], and they select the active microbial community and the conditions under which its enzymes work; the tannase of *A. parvulum*, for example, has its optimum at pH 6.0 [41]. Acidic beverages and sugar-induced plaque acidification are, therefore, expected to shift the balance between enzymatic conversion and spontaneous degradation.

*Diet.* Habitual diet shapes the oral community and, hence, its enzymatic capacity, as shown for the nitrate-reducing taxa (Section 2.6.1), and repeated polyphenol intake may itself select the community (Section 2.5.3). The food matrix also determines how long a polyphenol remains in the mouth: a beverage is cleared within minutes, whereas solid foods, chewing gums and lozenges release it slowly.

*Chewing and contact time.* Mastication and gustatory stimulation raise salivary flow from 0.3–0.4 to 1–2 mL/min [18], which accelerates clearance [25] and shortens contact time, but chewing also releases polyphenols from the food matrix and increases contact with the mucosa and the tongue dorsum. Holding a tea solution in the mouth without swallowing produced higher salivary catechin concentrations [10], and chewing green tea leaves delivered catechins to saliva at high concentrations [11]. Because oral conversion is expressed as a rate (Section 2.4.3), oral residence time is expected to matter as much as enzymatic capacity, and studies of oral biotransformation should report it.

#### 2.4.5. Proposal for an Operational Classification

Because discrete oral metabotypes cannot yet be defined (Section 2.4.3), we propose a provisional operational framework, termed oral hydrolytic capacity (OHC), constructed consistently with the observation in Section 2.4.3 that the oral phenotype is expressed through reaction rate. The classification is accordingly based on conversion kinetics measured at 1, 5, 15 and 30 min, expressed operationally as t_50_, the time required to convert half of the available substrate, as follows:

- OHC-0: conversion below the limit of quantification at all time points;
- OHC-L: t50 above 30 min;
- OHC-I: t50 between 5 and 30 min;
- OHC-H: t50 below 5 min.

**Figure 2:**
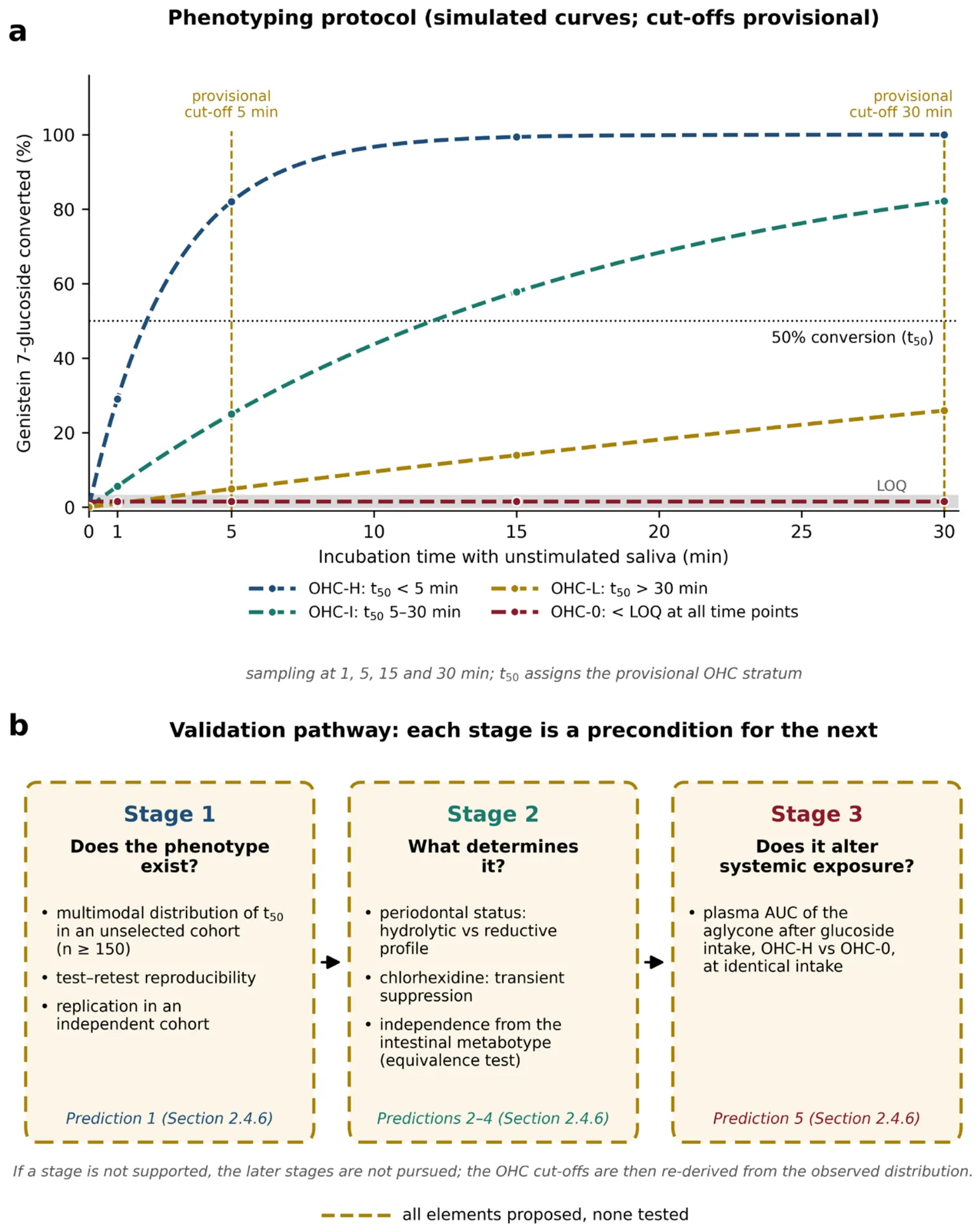
Proposed operational framework for the oral metabotype and the staged design required to test it. (**a**) Phenotyping protocol: A standard probe substrate, genistein 7-glucoside, is incubated with unstimulated saliva collected under recorded conditions; conversion is measured at 1, 5, 15 and 30 min, and the time to 50% conversion (t_50_) assigns the provisional oral hydrolytic capacity (OHC) stratum. The curves are simulated for illustration only, and the cut-offs are provisional anchors to be replaced by values derived from the observed distribution. (**b**) Validation pathway: stage 1 tests whether the phenotype exists (multimodality, reproducibility, and replication), stage 2 is what determines it, and stage 3 is whether it alters systemic exposure; each stage corresponds to the predictions in Section 2.4.6. All elements are proposals, and none has been tested.

The categories are mutually exclusive and cover the range completely. The proposed probe substrate is genistein 7-glucoside, for which reference human data and a known variability range already exist [8]. Alternatively, a chromogenic substrate of the fluorescein-di-β-D-glucoside type permits a rapid chairside test, a method already described on the lingual surface [9]. We state explicitly that this classification is a methodological proposal, not a result, and that the thresholds must be established empirically in a sufficiently large cohort. Until such data exist, OHC-0, OHC-L, OHC-I and OHC-H are to be read as strata of a measurement protocol awaiting validation rather than as biological categories, and the numerical cut-offs are provisional anchors chosen so as to be testable, not values derived from an observed distribution. Once such a cohort is available, the cut-offs should be derived from the observed distribution, for example, from the intersections of the components fitted by mixture modelling, rather than fixed in advance (Figure 2a; Section 2.8.2).

#### 2.4.6. Falsifiable Predictions

A conceptual framework has value only if it can be refuted. The present proposal generates five testable predictions.

1. Oral hydrolytic capacity is distributed multimodally rather than unimodally in an unselected cohort, the criterion that distinguishes a metabotype from a continuous gradient.
2. Patients with periodontitis show a qualitatively different conversion profile from periodontally healthy subjects, with a shift from hydrolytic towards reductive reactions.
3. Chlorhexidine rinsing transiently suppresses conversion capacity, with recovery correlated to recolonisation.
4. Oral hydrolytic capacity is independent of the intestinal metabotype of the same individual. Formulated rigorously, this is an equivalence hypothesis rather than an association hypothesis, and requires an equivalence test with a prespecified margin, for example, |r| below 0.20, together with an appropriate power calculation; failure to reject the null hypothesis of no correlation does not constitute evidence of independence.
5. In OHC-H subjects, the plasma area under the curve of the aglycone after glucoside intake is greater than in OHC-0 subjects, at identical intake.

The fifth prediction is the most consequential: if confirmed, the oral compartment would become a measurable determinant of systemic exposure to the aglycone, with implications beyond dentistry.

#### 2.4.7. Relationship with the Intestinal Metabotype

The comparison in Table 4 sets a concept validated in large cohorts against one still in the hypothesis stage. The asymmetry of status should not, however, be confused with an asymmetry of nature: the substantive differences concern the origin of the phenotype, the temporal window in which it is expressed and the parameter that must be measured to determine it.

The two are not competing but sequential. The oral compartment acts upstream of the intestinal one and modifies its substrate. An OHC-H individual delivers to the colon a partially deglycosylated substrate profile, different from that delivered by an OHC-0 individual. If the fourth prediction is confirmed, then the interindividual variability attributed until now exclusively to the gut microbiota has at least two sources.

### 2.5. The Reverse Direction: Remodelling of the Oral Community by Polyphenols

The preceding sections treated the oral microbiota as agent and the polyphenol as substrate. The perspective is reversed here. The final argument does not, however, consist in juxtaposing the two directions but in closing the loop: the community that biotransforms polyphenols is itself remodelled by them, and this remodelling presumably alters its biotransformation capacity.

#### 2.5.1. Three Distinct Mechanisms

The literature frequently treats the antimicrobial effect of polyphenols as undifferentiated. We distinguish three mechanisms with different ecological consequences: bactericidal or bacteriostatic, which reduces biomass and alters composition through selection; antivirulence, which alters the phenotype of the community without significantly altering its composition; and indirect ecological, which alters the host environment, redox, inflammation and matrix, with secondary selection of the community. Only the first changes who is present; the other two change what the present community does. For metabolic capacity, the latter two are probably the more relevant, although they have been far less studied.

#### 2.5.2. Antivirulence Without a Bactericidal Effect

The best-documented mechanism: Cranberry flavonoids, particularly A-type proanthocyanidins and myricetin, inhibit glucosyltransferase activity and exopolysaccharide-mediated adherence without killing the microorganisms [56]. A-type oligomers diminish insoluble glucan synthesis by GtfB adsorbed on saliva-coated hydroxyapatite and impair bacterial glycolysis, and in vivo the incidence of smooth-surface caries in rats was significantly reduced [54]. The mechanism extends to the transcriptional level: epigallocatechin gallate inhibits *Streptococcus mutans* biofilm formation by suppressing gtf genes [70].

This is an intervention on the genetic programme of the community, not on its viability. The exopolysaccharide matrix constitutes the scaffold of the biofilm, providing mechanical stability and facilitating the creation of localised acidic microenvironments [56]. Its disruption simultaneously alters the three-dimensional architecture, the pH and oxygen gradients and, implicitly, the available metabolic niches, and could, therefore, also alter the expressed enzymatic profile, a link that remains untested.

#### 2.5.3. Effects on Community Composition

Rinsing with a solution of epigallocatechin gallate effectively reduces the levels of mutans streptococci and lactobacilli in the oral cavity of children, the reduction being more pronounced for mutans streptococci than for lactobacilli [71]. The effect is, therefore, taxonomically selective rather than uniform, which means ecological selection, with a potential shift in the functional equilibrium of the community [72].

This selectivity raises an unexamined problem. If polyphenols preferentially suppress certain taxa, and the suppressed taxa are among those performing deglycosylation, then chronic polyphenol intake would progressively reduce oral bioactivation capacity, a mechanism of self-limitation of its own bioactivation. The hypothesis is counterintuitive and for precisely that reason deserves testing.

#### 2.5.4. Remodelling of the Host Matrix

Polyphenols physically alter the environment in which the biofilm develops. Epigallocatechin gallate substantially alters the properties of the MUC5B polymer network and of monomeric MUC7, producing a significant increase in mucin viscosity [20]. Increased viscosity of the mucin layer affects diffusion, clearance and bacterial adherence, an intervention on the niche rather than on its occupant, with direct implications for the design of mucoadhesive systems.

Similarly, epigallocatechin gallate precipitates salivary proteins and non-competitively inhibits α-amylase, limiting the formation of fermentable substrate, but the same study also documents the converse: α-amylase reduces the antimicrobial activity of epigallocatechin gallate against *A. actinomycetemcomitans* [12]. The interaction between polyphenol and salivary protein is, therefore, itself bidirectional, and the net outcome depends on the local stoichiometric ratio.

#### 2.5.5. Host Modulation as an Indirect Ecological Effect

Polyphenols intervene in the oral redox balance, with implications for dental caries, gingivitis, periodontitis and precancerous lesions, a level treated separately and in detail [24]. We retain here only the ecological consequence: potentiation of the oral peroxidases, which generate hypothiocyanite [23], constitutes a mechanism by which polyphenols alter the selective pressure on the community through the host, without direct contact with the bacteria.

At the inflammatory level, a randomised trial in 30 patients with chronic periodontitis showed that adding daily green tea consumption to scaling and root planing significantly reduces salivary IL-1β levels at six weeks, whereas the group treated by mechanical therapy alone showed no significant change [73]. The route of administration was ingestion, not rinsing, which means that exposure was simultaneously local and systemic: tea consumption generates salivary concentrations in the tens of micromolar range [10,11] and, in parallel, plasma exposure. The study, therefore, does not permit attribution of the effect to either route and illustrates precisely the interpretive problem formulated in Section 2.5.8. Irrespective of the mechanism, the reduction in inflammation alters the flow and composition of gingival crevicular fluid, and, thus, the protein substrate and physicochemical conditions of the subgingival niche (Section 2.1.5).

#### 2.5.6. Metabolites as Agents

A systematically omitted aspect is that the agent remodelling the community is not always the parent compound. Deglycosylation generates aglycones with markedly different physicochemical properties: removal of the bulky sugar moiety eliminates steric hindrance, introduces an additional hydroxyl group and facilitates transporter-independent cellular uptake [35]. A more lipophilic aglycone would be expected to cross the bacterial membrane and penetrate the biofilm more readily than the parent glycoside, although this has not been measured.

If this is the case, a consequence follows that, to our knowledge, has not been tested: The community may generate for itself the agent that remodels it. Bacteria that deglycosylate would produce aglycones with greater antibacterial activity than the precursor, creating a negative feedback loop in which the metabolic activity of the community limits its own expansion. The phenomenon is analogous to, but not identical with, bacteriocin production, since the substrate is exogenous.

Whether oral biotransformation increases or decreases biological activity has no single answer. The direction depends on the reaction, the substrate and the end point, and no study has compared the activity of a parent compound and its oral products in the same oral system. Deglycosylation generally increases lipophilicity and cellular uptake, and the aglycones quercetin and genistein inhibited the proliferation of oral cancer cells in vitro [8]; for local antiproliferative and antimicrobial end points this step is, therefore, expected to increase activity. For anthocyanins the gain is transient, because cyanidin degrades within hours at salivary pH [52]; the stable product, protocatechuic acid, was described as a functional metabolite [35], but it is a different molecule with a different activity profile. De-esterification of epigallocatechin gallate removes the galloyl group that contributes most to protein binding and to antibacterial activity [21,72], so this reaction is expected to reduce antimicrobial potency while increasing the free fraction. Phase II conjugation, if it occurs in oral mucosa, would be expected to reduce intrinsic activity, since glucuronidated and methylated conjugates are generally less active than the aglycones in vitro, although they may act as circulating forms that are deconjugated in tissues [2]. The net effect of oral biotransformation is, therefore, a change in the activity profile rather than a uniform increase or decrease, and it should be measured for each substrate rather than assumed.

#### 2.5.7. Synthesis

The five mechanisms described above are distinguished by their effect on two different things: who is present in the community and what the present community does (Table 5). The distinction matters, because the literature usually treats them together, under the single label of antimicrobial effect.

The last row is the hypothesis contributed by this section and, unlike the others, has not been tested. All the other mechanisms are individually documented, but none has been integrated into a recursive model. Figure 1b brings the two directions together in a single representation: the substrate enters the compartment, is transformed in parallel by the aerobic salivary phase and by the host epithelium, and the resulting products act locally, may pass into the circulation and, through the segment marked as untested, return upon the community that generated them.

#### 2.5.8. Consequence for the Interpretation of the Literature

The bidirectional model requires a revision of how intervention studies are interpreted. A trial administering a polyphenolic extract and measuring a periodontal parameter at six weeks cannot distinguish between the effect of the parent compound, the effect of locally generated metabolites, the effect of community remodelling and the effect of host modulation, four mechanisms with different therapeutic implications. We recommend that future studies include salivary metabolic phenotyping at baseline (Section 2.4.5) to permit stratification of results. The evidence-level coding in Figure 1b indicates where such attribution is currently possible and where it is not.

### 2.6. Local, Systemic and Pharmaceutical Consequences

An active metabolic compartment placed upstream of absorption alters both the local exposure to the active substance and the chemical identity of the species reaching the circulation. The argument is framed by a precedent in the same cavity, the nitrate–nitrite–nitric oxide axis, whose relevance and limits are set out below.

#### 2.6.1. The Demonstrated Precedent

The recycling of nitrate and nitrite to nitric oxide, known as the enterosalivary nitrate cycle, depends on commensal nitrate-reducing bacteria on the tongue dorsum, the most abundant being *Veillonella*, *Actinomyces*, *Haemophilus* and *Neisseria* [46]. The taxonomic attribution derives from metagenomic inventories and from direct testing of the reductive capacity of oral isolates [47,48]. Nitrite-producing species include representatives of the genera *Neisseria*, *Rothia*, *Veillonella*, *Actinomyces*, *Corynebacterium*, *Haemophilus* and *Kingella*, which constitute nearly 20% of the total genera measured in the oral cavity [74]. Nitrate and polyphenols are chemically unrelated, and the comparison is not mechanistic: nitrate reduction is a respiratory process carried out by dedicated reductases, whereas the polyphenol conversions discussed here are hydrolytic reactions on glycosidic and ester bonds. The precedent is used for a narrower purpose. It is the only case in which every step of the sequence considered in this review—an exogenous dietary substrate, conversion by the oral microbiota, a product with different biological activity and a measurable consequence that disappears when the oral flora is suppressed—has been demonstrated in humans. It, therefore, shows that such a sequence is physiologically possible and supplies a validated experimental design (Section 2.6.2); it does not show that the polyphenol sequence exists.

#### 2.6.2. The Intervention Evidence

The decisive demonstration came through pharmacological interruption of the microbial step. In a study of 19 healthy volunteers, with a seven-day control period followed by seven days of treatment with a chlorhexidine-based mouthwash, the treatment reduced oral nitrite production by 90% (*p* < 0.001) and plasma nitrite levels by 25% (*p* = 0.001), while systolic and diastolic blood pressure rose by 2–3.5 mmHg, the increases correlating with the fall in circulating nitrite concentrations (r^2^ = 0.56; *p* = 0.002) [75]. The effect on blood pressure appeared within one day of disruption of the oral flora and was sustained throughout the intervention [75].

The result has been replicated and extended. Chlorhexidine use attenuates the nitrate-reducing activity of oral bacteria by at least 80%, with the establishment of more acidic conditions and reduced nitrite availability [76]. In subjects on antihypertensive medication, attenuation of oral nitrate-reducing capacity after three days of daily chlorhexidine use was associated with a 2.3 mmHg rise in systolic pressure [77]. Earlier, the rise in plasma nitrite after a dietary nitrate load had been shown to be markedly attenuated by antibacterial mouthwash [78] and the gastroprotective and blood pressure-lowering effects of dietary nitrate to be abolished by antiseptic [79].

The significance of this body of evidence extends beyond cardiology: it establishes that a metabolic function of the oral microbiota can be suppressed by routine hygiene practices, with systemic consequences measurable within 24 h.

#### 2.6.3. Transfer of the Hypothesis to Polyphenols

Applying the same logic to the compartment described in Section 2.2, Section 2.3 and Section 2.4 yields a direct and testable hypothesis: oral antiseptics suppress the bioactivation capacity for polyphenolic compounds.

Preliminary data exist, obtained as an experimental control. The landmark study on salivary hydrolysis of flavonoid glucosides included an in vivo rinsing protocol: after a 30 s rinse with chlorhexidine or with Listerine, followed by a brief water rinse, saliva collected at successive intervals showed a significant reduction in the hydrolysis of genistein 7-glucoside relative to control [8]. The addition of antibiotics completely stopped the degradation of eugenyl-β-D-glucoside, confirming the microbiological origin of the hydrolysis [9].

What the authors used as evidence of the bacterial contribution can be read, from the perspective proposed here, as preliminary support for the hypothesis: a reduction in salivary hydrolysis after rinsing with antiseptics in current use has been observed in vivo in humans, in a single small experiment. What has never been assessed is the consequence: neither the plasma exposure to aglycones after suppression, nor any clinical endpoint. This is the distinction we formulate as a testing priority, with a design identical to that used for nitrate [75], substituting the substrate; the existence of pilot data [8] permits estimation of the effect size and of the required sample size.

The practical consequence is immediate and uninvestigated. A patient using chlorhexidine after periodontal therapy or an antiseptic mouthwash daily might fail to bioactivate polyphenolic compounds in the oral cavity, including those administered therapeutically for the very condition being treated. This remains a hypothesis. What has been demonstrated is a reduction in salivary hydrolysis after antiseptic rinsing in a small in vivo experiment [8]; the plasma, urinary and clinical consequences of that suppression have never been assessed, and the systemic consequence of chlorhexidine-associated suppression of salivary hydrolysis is, therefore, unknown.

#### 2.6.4. Polyphenols as an Alternative to Conventional Antiseptics

A convergence between the two axes deserves mention. Compared with chlorhexidine, propolis mouthwash had a smaller effect on the abundance of nitrite-producing species, and in the propolis group a significant increase in plasma nitrate (7 μmol/L) and a decrease in systolic pressure (2 mmHg) relative to baseline were observed [74]. A polyphenol-rich preparation, therefore, produces the opposite effect to chlorhexidine on this microbial function.

In parallel, rinsing with epigallocatechin gallate effectively reduces mutans streptococci and lactobacilli, and green tea and epigallocatechin gallate have been proposed as alternatives to chlorhexidine-based mouthwashes [71]. Bringing the two observations together, a different therapeutic profile of polyphenolic antiseptics emerges: selective suppression of pathogenic taxa with the preservation of the beneficial metabolic functions of the community, in contrast to the indiscriminate action of chlorhexidine. This distinction, if confirmed, constitutes a substantial pharmacological argument in favour of plant-derived antiseptics, different from the simplistic argument that a natural preparation is preferable by virtue of being natural.

There remains, however, a self-referential question that we formulate explicitly: does a polyphenolic antiseptic that preserves nitrate-reducing capacity also preserve deglycosylation capacity, that is, its own bioactivation, or does it on the contrary suppress it (Section 2.5.3)? The answer determines whether chronic use of such preparations is self-potentiating or self-limiting.

#### 2.6.5. The Oral Compartment as a Site of Presystemic Modification of the Chemical Species

The contribution of the oral compartment to systemic exposure is already documented in individual instances. Holding a solution of epigallocatechin gallate in the mouth generated the parent compound and epigallocatechin in saliva and, subsequently, epigallocatechin in urine [10], and saliva collected after rinsing contained glucuronidated anthocyanin conjugates [35]. These observations indicate that species formed or released in the mouth can subsequently appear systemically. They do not establish the size of that contribution: urinary epigallocatechin is compatible with de-esterification in the oral cavity but does not exclude gastrointestinal conversion of the swallowed fraction, and no study has quantified the proportion of systemic exposure attributable to oral biotransformation.

We formulate the consequence in pharmacokinetic terms. For flavonoid glycosides, the oral compartment is an underrecognised site of presystemic modification of the chemical species, positioned upstream of the intestinal and hepatic ones. Unlike these, there is no evidence that it reduces bioavailability but may alter it qualitatively, delivering aglycones rather than glycosides to absorption, with a different permeability and conjugation profile. Whether this amounts to a first-pass effect in the conventional pharmacokinetic sense, that is, one large enough to change systemic exposure, is not established by the present evidence and is examined quantitatively in the section titled The Quantitative Limit of the Oral Contribution. The physiologically based pharmacokinetic models developed for polyphenols to date do not contain this compartment.

##### The Quantitative Limit of the Oral Contribution

The above argument requires an order-of-magnitude assessment, absent from the literature to date. The residual salivary volume after swallowing is approximately 0.8 mL [25], the contact time per swallow is on the order of tens of seconds, and a 200 mL beverage is consumed within a few minutes. Even assuming complete conversion of the fraction in contact with the compartment at each cycle, the fraction of the ingested dose transformed orally plausibly remains below a few percent, against 12–48 h of colonic residence for the unabsorbed fraction. On this basis, the oral compartment does not, according to current evidence, meet the criterion for a quantitatively important first-pass effect, and no such claim is made here.

This finding does not invalidate the argument but delimits its domain of validity, on two levels. For local mucosal endpoints, oral chemoprevention, biofilm, periodontium, the relevant quantity is not the systemic mass balance but the concentration at the site, and there the oral compartment exceeds plasma by two orders of magnitude [10] and constitutes the dominant site of exposure. For topically applied oral dosage forms, where the entire dose is deposited in the compartment, the mass-balance restriction does not apply at all.

The oral mucosa is a permeable barrier, and transmucosal absorption constitutes a pharmaceutically exploited route of administration [80,81]. For systemic endpoints, the proposed effect is qualitative rather than quantitative: the compartment alters the identity of the chemical species delivered to absorption, not the total mass absorbed. A small mass fraction may become relevant if the resulting species has markedly superior permeability, which is the case for aglycones relative to their parent glycosides [35], and if direct oral mucosal absorption is efficient, a hypothesis supported by the detection of urinary epigallocatechin after holding a solution of epigallocatechin gallate in the mouth [10]. The fifth prediction in Section 2.4.6 tests precisely this alternative. Until it is tested, the contribution of locally generated metabolites to systemic exposure remains undetermined and is treated in this work as a research priority rather than as a finding.

#### 2.6.6. The Pathological Salivary Environment as a Determinant of Local Exposure

The proposed framework identifies clinical situations in which the oral compartment is profoundly altered, and these are precisely the situations in which topical oral therapy is most frequently indicated.

Xerostomia, whether from Sjögren syndrome, head and neck radiotherapy or anticholinergic polypharmacy, simultaneously alters several parameters of the compartment. Reduced salivary flow prolongs contact time and extends the biotransformation window (Section 2.1.3), but, at the same, time reduces clearance, alters protein composition and modifies the microbial community. The net effect on conversion and its magnitude are undetermined, but the consequence for formulation is direct: mucoadhesive systems designed on the basis of normal salivary parameters cannot be extrapolated to this environment. The saliva substitutes and artificial salivas used in these patients differ substantially from native saliva in protein composition, buffering capacity and rheology [82], which means that they also alter the availability of the free fraction of the active substance, an aspect not evaluated to date.

Periodontitis produces a presumed shift from the aerobic hydrolytic regime towards the anaerobic reductive one (Section 2.1.5), all the more relevant since periodontitis is itself the principal target of local polyphenol therapy. A preparation designed to act in the aerobic salivary compartment may encounter, at the target site, entirely different physicochemical conditions and enzymatic repertoire.

Complete edentulism and denture wearing radically alter community structure through loss of the supragingival niche. In the elderly, reduced salivary flow and altered microbiota composition are cumulative. In patients on antibiotic therapy, hydrolytic activity is directly suppressed [8,9]. In oral chemoprevention, the only context in which variability has already been correlated with therapeutic responsiveness [35], the proposed framework offers a mechanistic explanation.

#### 2.6.7. Implications for Topically Applied Oral Dosage Forms

The proposed framework has direct consequences for formulation. Polyphenolic compounds are not only dietary constituents but also active substances in mouthwashes, gels, varnishes and mucoadhesive systems intended for the supragingival biofilm and the periodontal pocket [83]. For these dosage forms, the compartment described here is not a transit stage but the medium of action proper, and local biotransformation determines the chemical identity of the substance that actually reaches the target.

The clinical precedent exists: bioadhesive black raspberry gels applied topically to premalignant oral lesions altered gene expression and loss-of-heterozygosity indices, and the intraoral distribution of the anthocyanins released from these formulations has been quantified in healthy volunteers [84,85,86]. The delivery strategies used in oral chemoprevention trials have been critically reviewed [87]. The consequence can be formulated as a principle: for a topically applied oral dosage form, the chemical species active at the site is not the one incorporated into the formulation but the product of the conversion performed by the compartment, and the identity of this product depends on the phenotype of the patient. If the classification proposed in Section 2.4.5 proves valid, a mucoadhesive system releasing a glycoside would deliver, in an OHC-H subject, the aglycone, and in an OHC-0 subject, the glycoside. The two differ in permeability, in affinity for salivary proteins and in antimicrobial profile. The choice between administering the glycoside and administering the preformed aglycone ceases to be a simple stability option and becomes a decision that determines whether the therapeutic effect depends on the patient’s phenotype. This principle is a consequence deduced from the proposed framework and not an observed result: no study has compared the performance of a topically applied polyphenolic dosage form among subjects stratified by oral conversion capacity.

To this is added the free-fraction problem (Section 2.1.2 and Section 2.7.4). The total concentration measured by extraction systematically overestimates the pharmacologically active fraction, and the deviation becomes critical in the pathological salivary environment, where protein composition and flow are altered. We recommend that in vitro release studies for topically applied oral dosage forms report the free fraction in human saliva or in a simulated medium with realistic protein composition, not merely the total amount released.

#### 2.6.8. Local Interactions Between Phytochemicals and Medication

The immunohistochemically documented presence in the oral mucosa of UDP-glucuronosyltransferases 1A, catechol-O-methyltransferase and the breast cancer resistance protein efflux transporter [35] opens a question not discussed to date: that of local interactions. What follows is a mechanistic hypothesis, advanced in the absence of interaction data of any kind. Polyphenols are documented inhibitors of efflux transporters and phase II enzymes in other compartments, and the logic of interaction between plant-derived compounds and medication through enzymes and transporters is well established systemically [6]. Co-administration of a topically applied polyphenolic preparation with a dosage form absorbed buccally or sublingually, therefore, constitutes a plausible and entirely uninvestigated interaction scenario. We formulate this observation as a hypothesis rather than as an established fact: no measurement of BCRP or UGT1A inhibition has been performed on human oral mucosal tissue. No in vivo or clinical study has examined whether a topically applied polyphenolic preparation alters the absorption of a buccally or sublingually administered drug, and no clinical interaction potential should be inferred from this section. On the scale used in Section 2.6.9, this scenario belongs to the lowest evidence tier.

#### 2.6.9. Synthesis

The two axes can be compared element by element, from substrate to the effect of microbial suppression (Table 6). The right-hand column contains three cells marked as untested for which the left-hand column provides complete answers. These are, in essence, the agenda detailed in Section 2.8.2.

The pharmaceutical implications discussed in Section 2.6.3, Section 2.6.4, Section 2.6.5, Section 2.6.6, Section 2.6.7 and Section 2.6.8 differ widely in the strength of their support and are graded here on the three-tier scale used throughout (Section 3). Three points are supported by in vivo human data: salivary exposure depends on the dosage form and is absent when the same polyphenols are given in capsules [10]; a single antiseptic rinse reduces the salivary hydrolysis of a flavonoid glucoside [8]; and mucoadhesive black raspberry formulations deliver anthocyanins to the oral mucosa and produced local molecular and histological effects in clinical studies [84,85,86]. Two points are supported only by ex vivo, in vitro or expression data: the reduction in the free fraction by salivary proteins [12,20] and the presence in oral mucosa of enzymes and transporters that could conjugate or efflux polyphenols [35]. Three points have no direct evidence and remain hypotheses: the systemic consequences of antiseptic suppression (Section 2.6.3), a dependence of mucoadhesive formulation performance on the oral conversion phenotype of the patient (Section 2.6.7), and local interactions involving UGT1A, COMT or BCRP with buccally or sublingually administered drugs (Section 2.6.8), which rest on protein expression and on analogy with other tissues. Only the first group can inform current practice; the other two define research questions.

### 2.7. Methodological Considerations

The gaps identified are explained not by lack of interest but by the absence of an adequate methodological framework. The oral compartment imposes constraints that the toolkit developed for the gut microbiota does not cover time windows on the order of minutes, small sample volumes, a community organised in adherent biofilm rather than in suspension, and a permanent overlap between microbial and host metabolism.

#### 2.7.1. Ex Vivo Models with Whole Saliva

These are the models that have generated almost all existing data [8,9,10,35,38]. The advantages are evident: a non-invasive sample, a real human community, a simple set-up. The limitations, however, are systematic and underestimated.

The first is loss of biofilm structure. Saliva contains predominantly bacteria detached from biofilms, in suspension, and the adherent community, which represents the majority of the biomass and has a distinct transcriptional profile, is not represented. These cells are not, however, equivalent to a planktonic laboratory culture either, since they originate from a biofilm and retain part of its physiological state. Extrapolation from saliva to biofilm is not validated in either direction.

The second is incubation duration, in our view the principal source of the discrepancies in the literature. A 60 min incubation measures a different phenomenon from one lasting several days. The observation that bacterially derived glycosidase activity in saliva becomes significant only after several days of reaction [38] requires that any study of oral biotransformation report kinetics rather than endpoints.

The third is confusion between host and microbiome. Discrimination requires antibiotic controls [8,9], filtration, or comparison between whole and microflora-reduced saliva [35], and not all studies include them.

The fourth is absence of sampling standardisation: stimulated or unstimulated saliva, time of collection, interval since the last meal and since the last toothbrushing, and the donor’s periodontal status are variables that directly affect the result and are reported inconsistently [88].

#### 2.7.2. Oral Biofilm Models

In vitro oral biofilm models, constant-depth film fermenters, continuous-flow systems and multispecies models on saliva-coated hydroxyapatite supports [89] are well established in cariology. They have been used extensively to study the effects of polyphenols on the biofilm [54,55,56] but never to measure biotransformation of polyphenols by the biofilm.

The necessary instrument already exists and is validated; it has not been turned in the opposite direction. An experiment in which a multispecies biofilm derived from human saliva is exposed to a flavonoid glycoside, with LC-MS/MS quantification of the aglycone in the supernatant and in the matrix, is immediately feasible with modest resources.

#### 2.7.3. In Vivo and In Situ Models

Three approaches are applicable in humans. Rinsing protocols with serial saliva sampling have been used successfully for catechins [10,11] and anthocyanins [35] and are the de facto standard for local oral pharmacokinetics. Removable intraoral devices carrying a biofilm substrate permit in situ exposure and recovery for analysis. Finally, rapid tests on the mucosal surface: the described method using fluorescein-di-β-D-glucoside applied on filter paper to the lingual surface, with detection of released fluorescein under ultraviolet light within 15–20 s [9], constitutes a prototype chairside test for the phenotyping proposed in Section 2.4.5. It is, to our knowledge, the only published method for rapid in vivo assessment of oral hydrolytic capacity, and its potential has not been exploited.

#### 2.7.4. Analytics and the Free-Fraction Requirement

LC-MS/MS is the reference method [9,10,35]. The oral compartment imposes, however, three specific requirements. The first is sensitivity in small volumes and in a matrix with high protein content, dominated by mucins, which requires adapted deproteinisation protocols validated according to ICH Q2(R2).

The second, and most important, is discrimination of the free fraction from the protein-bound fraction. Given that epigallocatechin gallate binds α-amylase with Kd = 2.74 × 10^−6^ M [12] and precipitates several salivary proteins and that mucins alter their rheology in its presence [20], the extracted total concentration systematically overestimates the pharmacologically active fraction. Ultrafiltration or equilibrium dialysis should become obligatory steps in determining salivary exposure by analogy with standard practice in plasma pharmacokinetics. This is, in our view, the most important methodological recommendation of the present work and the easiest to implement.

The third is preanalytical stability: Anthocyanins undergo non-enzymatic degradation in saliva [49,52]; the near-neutral pH of the flavylium cation is hydrated to the colourless hemiketal, which opens to *cis*- and *trans*-chalcones, and these undergo C-ring cleavage to a phenolic acid and an aldehyde, protocatechuic acid and phloroglucinaldehyde in the case of cyanidin, accompanied by oxidative degradation; the rates increase with the pH and temperature and are higher for aglycones than for glycosides [52], and samples require immediate acidification and freezing. Catechins raise a symmetrical problem: epigallocatechin gallate autoxidises and epimerises rapidly at neutral pH and room temperature, forming dimers and epimers that may be mistaken for biotransformation products [90,91]. In the absence of these precautions, spontaneous degradation of the aglycone is erroneously reported as metabolism.

Untargeted salivary metabolomics offers a complementary approach, with known limitations: the difficulty of establishing direct associations between specific metabolites and mechanisms, considerable interindividual variability and the complexity of the oral microenvironment [92]. The latter two are, from the perspective of the present work, precisely the objects of study.

#### 2.7.5. Community Characterisation and Functional Attribution

Resolving the gap identified in Section 2.2.6 requires going beyond 16S sequencing, which provides composition rather than function. The appropriate toolkit comprises shotgun metagenomics, for an inventory of the genes encoding glycoside hydrolases and esterases; metatranscriptomics, to distinguish genes present from genes expressed; and isolation with strain-level validation as the final step, by analogy with the identification of intestinal urolithin producers [4,5] and with the approach already applied successfully to PsBG1 and to the two oral tannases [40,41,42]. This sequence is exactly the one traversed over the past fifteen years for the gut microbiota. For the oral microbiota it has barely begun.

#### 2.7.6. Proposed Minimum Standards

We propose that future studies of the oral biotransformation of polyphenolic compounds report as mandatory the type of saliva, stimulated or unstimulated, and the measured flow rate and time of collection; the periodontal status of the donors, with, at minimum, the bleeding on probing index and probing depths; the interval since the last meal, the last toothbrushing and the last mouthwash use; the complete kinetics of conversion, with, at minimum, three time points within the first 30 min; the controls discriminating between host and microbiome; the free fraction compared with the total analyte; and the number of donors, together with the individual distribution of the results, not only the mean, given that variability is, in this field, the signal rather than the noise.

An additional, cross-cutting requirement, absent from the entire existing literature, deserves separate formulation. Attributing a transformation to the oral compartment requires excluding the subsequent digestive stages as an alternative source of the same product, since several of the reactions discussed here, glycosidic hydrolysis, de-esterification, non-enzymatic degradation of aglycones, also occur in the stomach or small intestine. In vitro models simulating gastrointestinal conditions, already applied to confirm the biochemical stability of the phenolic fractions of sprouted buckwheat-based matrices [93], provide the necessary control: parallel incubation of the same substrate with human saliva and with simulated gastric and intestinal fluids permits separation of oral conversion from that of the subsequent stages and quantification of the relative contribution of each. None of the oral studies cited in this synthesis included such a control, which means that the compartment specificity of the documented reactions remains, strictly speaking, assumed.

The suitability of the available experimental models for this question is compared in Table S2. Three of the most appropriate among them have never been applied to the question at hand. This, rather than any intrinsic technical difficulty, explains the current state of the field.

### 2.8. Limitations and Research Agenda

#### 2.8.1. Limitations of the Present Synthesis

We state the limitations of this work explicitly, since part of its construction is a proposal rather than evidence synthesis. The first is methodological. This is a narrative synthesis, not a systematic review. We did not apply a registered protocol, predefined eligibility criteria or formal risk-of-bias assessment. The choice is justified by the extreme dispersion of the literature, but the consequence is real: we cannot exclude the omission of relevant studies, particularly from the flavour perception literature.

The second concerns the balance of proposal against evidence. Section 2.1, Section 2.2, Section 2.3, Section 2.6.1 and Section 2.6.2 are a synthesis of published evidence. Section 2.4 on oral metabotypes, Section 2.5.6 on the loop of self-generation of the remodelling agent, Section 2.2.5 and Section 2.3.5 on the subgingival reductive regime and oral urolithin production, Section 2.6.3 on the systemic consequences of antiseptic interference and Section 2.6.8 on local interactions are hypotheses formulated by the authors, supported by analogy and indirect evidence. The OHC classification proposed in Section 2.4.5 belongs to the same category: it is a measurement protocol with provisional thresholds, not a validated phenotype. We have marked this distinction in the text and reiterate it here to prevent their being cited as established facts.

The third concerns the evidence base, which is thin. The key studies are few and small: 17 subjects for the quantification of hydrolytic variability [8], 6 volunteers for salivary catechin pharmacokinetics [10], 15 mucosal tissues for enzymatic characterisation [35]. None has been independently replicated on a larger scale, and some are more than two decades old.

Confidence in the oral metabotype is correspondingly low. The available data show that oral conversion capacity varies among individuals, but variability of this size in 17 subjects is equally compatible with a continuous distribution and does not demonstrate discrete phenotypes. The concept should, therefore, be regarded as hypothesis-generating: it is consistent with the available observations, no study designed to test it has been performed, and its central prediction, multimodality, could fail. The design that would provide the strongest test is described in Section 2.8.2.

The fourth is the risk of over-extrapolation from analogy. The argument in Section 2.6 rests on the parallel with the nitrate–nitrite–nitric oxide axis. The analogy is structural rather than mechanistic: nitrate reduction is a central respiratory function for the taxa performing it, whereas glycoside hydrolysis is probably an accessory activity. There is no guarantee that a mechanism valid for the former transfers to the latter, and the quantitative limit discussed in the section titled The Quantitative Limit of the Oral Contribution remains an open objection for systemic endpoints.

The fifth concerns the existing molecular characterisation. The three characterised oral enzymes [40,41,42] were studied as recombinant proteins on model substrates that do not include dietary polyphenols. The presence of the gene and the activity of the purified enzyme do not demonstrate activity in situ in the human biofilm.

The sixth concerns the quantitative reach of the framework. The evidence assembled here supports local oral biotransformation and presystemic modification of the chemical species; it does not support a first-pass effect in the conventional pharmacokinetic sense. The mass-balance estimate in the section titled The Quantitative Limit of the Oral Contribution places the orally transformed fraction of an ingested dose plausibly below a few percent, the direct transmucosal contribution to systemic exposure is unquantified, and no study has separated orally formed from gastrointestinally formed metabolites in the systemic pool. On the present evidence, the framework is, therefore, primarily one of local and qualitative consequence, and its systemic implications are stated as predictions.

Finally, two contradictions remain unresolved: the status of rutin as a substrate [8] versus the findings reporting in [35,49], and the discrepancy regarding the time required for bacterial glycosidase activity [8,9] versus the findings reporting in [38]. We have not been able to arbitrate them on the basis of the existing literature. These limitations bear directly on the strength of the conclusions and are reflected in Section 4.

#### 2.8.2. Research Agenda

The research directions follow from the gaps identified above and are ordered by the ratio between immediate feasibility and potential impact; the nine objectives, with the proposed design, estimated feasibility and the prediction each tests, are summarised in Table S3. Four studies are feasible immediately and with modest resources. The first, which should precede all others, characterises the population distribution of oral hydrolytic capacity in a cohort of at least 150 subjects, using a standardised probe substrate (genistein 7-glucoside), kinetics at 1, 5, 15 and 30 min and LC-MS/MS quantification; it tests the first prediction in Section 2.4.6 and thereby validates or refutes the concept of the oral metabotype. The second resolves the rutin contradiction through parallel incubations of rutin and isoquercitrin in the same donors, at least 30, at 5, 30 and 60 min and at 24 h, with reporting of the individual distribution. The third replicates the design used for nitrate [75] with a polyphenolic substrate, in a seven-day crossover study in 20 subjects that measures oral conversion and plasma exposure to aglycones during chlorhexidine use. The fourth determines the free fraction of the principal classes in human saliva by ultrafiltration coupled with LC-MS/MS, in healthy and in xerostomic subjects.

In the medium term, enzyme–taxon attribution for polyphenolic substrates can be pursued by shotgun metagenomics and metatranscriptomics of salivary samples correlated with the metabolic phenotype, followed by isolation and strain-level validation. The validated multispecies biofilm models [54,55,56] can be used to quantify polyphenol conversion rather than only its effects on the biofilm. The subgingival reductive regime can be tested by anaerobic incubation of subgingival plaque with ellagic acid and with (+)-catechin, with LC-MS/MS detection of urolithins and phenyl-γ-valerolactones, which we regard as the question with the greatest potential consequence (Section 2.3.5). The oral and intestinal metabotypes of the same individuals can then be compared by an equivalence test, as required by the fourth prediction.

Long-term objectives are the stratification of intervention trials by baseline oral metabolic capacity, the incorporation of the oral compartment into physiologically based pharmacokinetic models, the development of antiseptic preparations that preserve the beneficial metabolic functions of the community, starting from the profile observed for propolis [74], and the assessment of the impact of the oral metabotype on the performance of mucoadhesive polyphenol systems.

The strongest evidence for or against the oral metabotype would come from a staged design (Figure 2b). The first stage is a prospective cohort of unselected adults large enough for mixture modelling, on the order of 150 to 300 subjects, phenotyped with a standardised probe substrate under fixed sampling conditions, with repeated measurement in a subset after two to four weeks to establish test–retest reliability. A metabotype is supported only if the distribution is multimodal by a prespecified criterion, if individuals keep their category on repeated measurement and if the result is replicated in an independent cohort. The second stage links the phenotype to its cause, by shotgun metagenomics of the same samples and by a randomised crossover in which chlorhexidine is used to suppress the microbial component. The third stage tests pharmacological relevance, through a crossover pharmacokinetic study in which subjects at the two extremes of the distribution receive the same glucoside dose and plasma exposure to the aglycone is compared (fifth prediction). A positive first stage would establish the phenotype; only the third would establish its importance for systemic exposure.

## 3. Materials and Methods

This is a narrative synthesis, reported in accordance with the SANRA recommendations for narrative reviews [94]. The format was chosen deliberately, because the relevant literature is dispersed across fields with non-convergent terminologies, pharmacokinetics, oral microbiology, food science and sensory research, preventive oncology and dentistry, and a single systematic search strategy cannot cover these vocabularies simultaneously. The objective was not to estimate an aggregate effect but to reconstruct a conceptual framework and to identify gaps explicitly.

PubMed/MEDLINE, Scopus, Web of Science and Google Scholar were searched from database inception to 31 August 2026, the date of the final re-run of all searches. Terms were combined in four blocks: (i) polyphenol*, flavonoid*, catechin*, anthocyanin*, proanthocyanidin*, ellagitannin*, phenolic acid*; (ii) oral cavity, saliva*, oral microbio*, dental biofilm, dental plaque, gingival crevicular fluid; (iii) metaboli*, biotransformation, deglycosylation, hydrolysis, glycosidase, esterase, tannase, bioactivation; (iv) metabotype, interindividual variability, bioavailability, presystemic metabolism. Blocks (i) to (iii) were combined with the AND operator to form the core search. Block (iv) was not added to the core search with AND, because the metabotype and interindividual variability literature rarely indexes oral terms and would have been lost; it was combined with block (i) in a separate, supplementary search. Records from both searches were merged before deduplication, and truncation operators were adapted to each database.

The PubMed/MEDLINE string was: (polyphenol*[tiab] OR flavonoid*[tiab] OR catechin*[tiab] OR anthocyanin*[tiab] OR proanthocyanidin*[tiab] OR ellagitannin*[tiab] OR phenolic acid[tiab]) AND (oral cavity[tiab] OR saliva*[tiab] OR oral microbio*[tiab] OR dental biofilm[tiab] OR dental plaque[tiab] OR gingival crevicular fluid[tiab]) AND (metaboli*[tiab] OR biotransformation[tiab] OR deglycosylation[tiab] OR hydrolysis[tiab] OR glycosidase[tiab] OR esterase[tiab] OR tannase[tiab] OR bioactivation[tiab]), with no language or date filter applied at the search stage. This core string corresponds to blocks (i) to (iii). The supplementary search combined block (i) with block (iv) (metabotype, interindividual variability, bioavailability, presystemic metabolism), using the same field tags, in order to capture the metabotype literature that does not index the oral compartment; the core and supplementary searches are, therefore, complementary rather than nested. Equivalent strings, adapted to the syntax of each interface, were used in Scopus (TITLE-ABS-KEY) and Web of Science (Topic); in Google Scholar, the first 200 records per combination, sorted by relevance, were screened.

In vitro, ex vivo, animal and human in vivo studies were included, as were previous syntheses, published in English, reporting interactions between polyphenols and the oral compartment. Works from the sensory perception literature documenting oral microbial hydrolysis of glycosidic precursors were also retained, since these constitute relevant mechanistic evidence even where their stated objective differed. Reference lists of included articles were screened manually, and the citing articles of key studies were checked.

Titles and abstracts were screened by all authors, and any record considered potentially relevant by either was retrieved in full. Full texts were assessed against the criteria above. Records were excluded when the oral cavity appeared only as a route of administration without measurement in that compartment, when polyphenols were treated solely as analytical or antimicrobial agents without reference to biotransformation, and when no primary or clearly attributed data were reported. Backward screening of reference lists and forward citation screening were performed for the studies that anchor the argument [8,9,10,11,35,38,74,75]. Because the objective was conceptual reconstruction rather than the aggregation of an effect estimate, no registered protocol, no formal risk-of-bias instrument and no quantitative synthesis were applied; the three-tier evidence grading described below serves this function, and the resulting classification of every source used is displayed in Table 1, Table 2, Table 3, Table 4, Table 5 and Table 6 and in the Supplementary Materials.

The searches of the three indexed databases retrieved 2115 records (PubMed/MEDLINE 422, Scopus 1005, and Web of Science 688), and 19 further records were identified from reference lists and citation tracking. After removal of 901 duplicates by automated matching of DOI and normalised title, 1233 records were screened by title and abstract, 103 full texts were assessed and 68 were excluded, most often because they addressed polyphenol–salivary protein binding in the context of astringency, a topic covered here through syntheses [21,22]. The synthesis is based on the 35 studies retained at the full-text stage; the remaining references provide background on physiology, analytical methods and pharmacokinetics.

Data were organised by anatomo-functional compartment and by substrate class, and the level of evidence was graded on three tiers: documented in vivo in humans, documented exclusively in vitro or ex vivo, and undocumented, formulated as hypothesis. This grading is marked explicitly in the tables in the text and in the Supplementary Material. The authors’ own hypotheses are identified as such in the text and listed in Section 2.8.1.

## 4. Conclusions

The oral cavity is the first site at which ingested or locally applied polyphenols meet enzymes able to transform them. Published evidence shows that, within minutes of contact with saliva, flavonoid glucosides are deglycosylated, anthocyanins are deglycosylated and degraded to protocatechuic acid, and epigallocatechin gallate is de-esterified, at local concentrations about two orders of magnitude above those in plasma. Of the enzymes expressed in oral mucosa, only β-glucosidase has confirmed activity.

These reactions change the chemical form of the compounds presented to the mucosa and to absorption. The fraction of an ingested dose converted in the mouth is small, and a first-pass effect large enough to alter systemic pharmacokinetics has not been shown; the established consequences are local and qualitative.

The large interindividual differences in conversion capacity, reported in small studies, led us to propose the oral metabotype and an operational classification based on oral hydrolytic capacity. Both are hypotheses. Testing them requires adequately sized cohorts with standardised phenotyping, assessment of reproducibility and pharmacokinetic follow-up. Until such data exist, the systemic effects of antiseptic suppression, a phenotype-dependent performance of oral dosage forms and local interactions with buccally or sublingually administered drugs should be treated as research questions, not as a basis for clinical recommendations.

## Figures and Tables

**Table 1:** The oral compartment compared with the colonic compartment as sites of microbial biotransformation of polyphenols.

Parameter	Oral Cavity (Salivary Phase)	Gingival Sulcus/Pocket	Colon
Residence time	seconds–minutes; salivary t_1/2_ 10–20 min [10]	hours–permanent (adherent biofilm); not measured	12–48 h [32]
Measured local concentration	2–145 μM (catechins, after tea consumption) [10,11]	undetermined	micromolar–millimolar (estimated) [33]
pH	6.2–7.6 [18]	6.9–8.0 (rises in periodontitis) [31]	5.5–7.0 [34]
Redox potential (E_h_)	+200/+300 mV [15]	−100/−300 mV [15]	−200/−300 mV [15]
Oxygenation	aerobic/microaerophilic [15]	anaerobic [15]	strictly anaerobic [15]
Bacterial density	10^8^–10^9^/mL saliva [15]	10^11^/g plaque [15]	10^11^–10^12^/g content [32]
Documented reactions	hydrolysis: deglycosylation, de-esterification [8,9,10]	none documented in situ	reduction, dehydroxylation, decarboxylation, C-ring cleavage [2,3]
Host phase II metabolism	proteins expressed (UGT1A, COMT, SULT, BCRP, immunohistochemistry); function confirmed only for β-glucosidase [35]	undetermined	present (enterocyte)
Enzyme–taxon attribution	partial: tannases, one β-glucosidase (Section 2.2.6)	absent in situ	achieved for several pathways [4,5]
Modifiability by intervention (Section 2.6)	high (hygiene, antiseptics, diet, salivary flow)	high (periodontal therapy)	moderate (diet, probiotics)

**Table 2:** The enzymatic repertoire of the oral compartment relevant to polyphenol biotransformation, with nitrate reductase as a reference activity.

Enzyme (EC)	Documented Substrate	Product	Origin	Level of Evidence
β-Glucosidase (3.2.1.21)	quercetin 4′-glucoside, genistein 7-glucoside	quercetin, genistein	bacterial and epithelial	in vivo human [8]
β-Glucosidase	black raspberry anthocyanins	cyanidin, then protocatechuic acid	whole and microflora-reduced saliva	in vivo human [35]
β-Glucosidase	eugenyl-β-D-glucoside	eugenol	bacterial (confirmed by antibiotics)	in vitro and in vivo [9]
β-glucosidase PsBG1	aroma glucosides, chromogenic substrates	odorant aglycones	*Prevotella* sp. (recombinant)	in vitro, structure solved [40]
β-Glycosidases	glycosidic aroma precursors	odorant aglycones	bacterial, species-dependent	in vitro, human saliva [38,39]
Lactase-phlorizin hydrolase	flavonoid glycosides	aglycones	epithelial (basal/spinous layer, salivary ducts)	immunohistochemistry [35]
Catechin esterase (unspecified)	epigallocatechin gallate	epigallocatechin	salivary, origin unspecified	in vivo human [10]
Tannase TanA_Ap_ (3.1.1.20)	methyl gallate	gallic acid	*Atopobium parvulum* (recombinant)	in vitro; 3.5 U/mg [41]
Tannase TanB_Fnp_ (3.1.1.20)	methyl gallate	gallic acid	*F. nucleatum* subsp. *polymorphum* (recombinant)	in vitro; 699 U/mg [42]
UGT1A/COMT/SULT/BCRP	aglycones	glucuronide, methylated conjugates	epithelial	immunohistochemistry and LC-MS/MS [35]
Nitrate reductase ^a^	nitrate	nitrite	bacterial (*Rothia*, *Actinomyces*, *Veillonella*)	in vivo human [46,47,48]
Reductases/decarboxylases	n.s.	n.s.	hypothesis (subgingival biofilm)	none (hypothesis)

n.s., not studied. ^a^ Nitrate reductase does not act on polyphenols; it is included as a reference activity, the only oral microbial biotransformation for which the responsible taxa, the in vivo activity and a systemic consequence are all established (Section 2.6.1).

**Table 3:** Degree of characterisation of the polyphenol classes in the oral compartment.

Class	Documented Oral Reaction	Product	In Vivo Human Evidence	Correlation with Donor Microbiota
Flavonoid glucosides	selective deglycosylation [8]	aglycones	yes	no
Anthocyanins	deglycosylation, spontaneous degradation, phase II [35,49,52]	cyanidin → protocatechuic acid; glucuronides	yes	no
Galloylated catechins	de-esterification [10]	epigallocatechin	yes	no
Volatile phenolic glycosides	deglycosylation [38,39,40]	odorant aglycones	in vitro, saliva	partial (bacterial dependence)
Proanthocyanidins	none documented; effects on oral biofilm only [54,55,56]	n.s.	n.s.	n.s.
Ellagitannins and ellagic acid	none documented ^a^	n.s.	n.s.	n.s.
Hydroxycinnamic acids	none documented ^a^	n.s.	n.s.	n.s.

n.s., not studied. ^a^ No study of this class in saliva, oral biofilm or oral mucosa was identified (a total gap in the literature); the entry indicates absence of data, not a negative result.

**Table 4:** Intestinal metabotype compared with the proposed oral metabotype.

Characteristic	Intestinal Metabotype	Oral Metabotype (Proposed)
Status	established, validated in large cohorts [3,5]	hypothesis, with three lines of indirect evidence (Section 2.4.2)
Defined examples	UM-0/A/B, equol, lunularin [5,67,68]	OHC-0/L/I/H, proposed (Section 2.4.5)
Origin	exclusively microbial [3]	mixed: microbial and epithelial [8,35]
Temporal window	hours to days [3,5]	minutes [8,10]
Measured parameter	final yield (urine, faeces) [5,69]	conversion rate (saliva) (Section 2.4.5)
Phenotyping matrix	urine, faeces [5,69]	saliva [8,9]
Modifiability	moderate [3]	high (Section 2.4.4)
Demonstrated relevance	cardiovascular, metabolic, oncological [61,69]	oral oncology, indirectly [35]

**Table 5:** Mechanisms of remodelling of the oral compartment by polyphenols.

Mechanism	Effect on Composition	Effect on Phenotype	Documented Examples	Presumed Impact on Metabolic Capacity
Glucosyltransferase inhibition, antivirulence	minimal	major	A-type proanthocyanidins, myricetin, EGCG [54,55,56,70]	indirect, through biofilm architecture
Selective bactericidal	major	not assessed	EGCG vs. *S. mutans*, lactobacilli [71,72]	direct, through loss of hydrolytic taxa
Modulation of the salivary matrix	not assessed	not assessed	interaction with MUC5B and α-amylase [12,20]	diffusion, adherence, fermentable substrate
Potentiation of host defence	indirect	indirect	oral peroxidases [23,24]	host-mediated selective pressure
Modulation of inflammation	indirect	indirect	green tea, decrease in salivary IL-1β [73]	alters crevicular fluid and subgingival niche
Locally generated aglycones	unknown	unknown	cyanidin, quercetin, genistein [8,35]	feedback loop, untested

**Table 6:** Parallels between the demonstrated nitrate–nitrite–nitric oxide axis and the proposed polyphenol–aglycone axis.

Element	Nitrate Axis (Demonstrated)	Polyphenol Axis (Proposed)
Substrate	nitrate (leafy vegetables) [46]	flavonoid glycosides, galloylated catechins [1]
Oral microbial reaction	nitrate reduction to nitrite [46,47]	hydrolysis: deglycosylation, de-esterification [8,10]
Identified taxa	*Neisseria*, *Rothia*, *Veillonella*, *Actinomyces* and others (~20% of genera) [46,47,48]	tannases: *A. parvulum*, *F. nucleatum*; β-glucosidase: *Prevotella* sp.; none correlated with phenotype [40,41,42]
Product with superior activity	nitrite, then nitric oxide [75]	aglycones; higher potency shown in vitro only [35]
Demonstrated systemic effect	blood pressure reduction in 2–3.5 mmHg [75,77]	untested
Effect of microbial suppression	chlorhexidine: −90% oral nitrite, −25% plasma, blood pressure rise [75,76,78,79]	chlorhexidine and Listerine: salivary hydrolysis significantly reduced in vivo [8]; plasma and clinical consequence untested
Interindividual variability	documented [48]	documented, more than twenty-fold [8]
Modulation by polyphenolic preparations	propolis preserves the function, unlike chlorhexidine [74]	untested

## Data Availability

No new data were created or analyzed in this study. Data sharing is not applicable to this article.

## Supplementary Materials

The following supporting information can be downloaded at: https://www.mdpi.com/article/10.3390/ph19101661/s1. Table S1: Candidate determinants of the proposed oral metabotype, formulated as testable hypotheses; Table S2: Suitability of the available models for the study of oral biotransformation of polyphenols; Table S3: Prioritised research agenda.

## Abbreviations

The following abbreviations are used in this manuscript:

BCRP	Breast cancer resistance protein
COMT	Catechol-O-methyltransferase
C_max_	Maximum concentration
CYP	Cytochrome P450
EC	Enzyme Commission number
EGCG	(−)-Epigallocatechin gallate
E_h_	Redox potential
GtfB	Glucosyltransferase B of *Streptococcus mutans*
ICH	International Council for Harmonisation
IL-1β, IL-6	Interleukin 1 beta, interleukin 6
K_d_	Dissociation constant
LC-MS/MS	Liquid chromatography–tandem mass spectrometry
MUC5B, MUC7	Salivary mucins 5B and 7
OHC	Oral hydrolytic capacity (OHC-0, OHC-L, OHC-I, OHC-H)
PsBG1	β-Glucosidase 1 of oral *Prevotella* sp.
SANRA	Scale for the Assessment of Narrative Review Articles
SULT	Sulfotransferase
TanA_Ap_	Tannase A of *Atopobium parvulum*
TanB_Fnp_	Tannase B of *Fusobacterium nucleatum* subsp. *polymorphum*
TNF-α	Tumour necrosis factor alpha
t_1/2_	Elimination half-life
t_50_	Time to 50% substrate conversion
UGT1A	UDP-glucuronosyltransferase family 1 member A
UM	Urolithin metabotype (UM-0, UM-A, UM-B)
