# Supplementary material for: The Forgotten First Contact: The Oral Cavity as a Site of Local Biotransformation Shaping the Chemical Form of Polyphenolic Drugs and Dietary Compounds
Source: Pharmaceuticals (Basel). 2026 Oct 21;19(10):1661. doi: 10.3390/ph19101661 (PMC13708039; doi:10.3390/ph19101661)
Supplement: Supplementary file 1 [file pharmaceuticals-19-01661-s001.zip › pharmaceuticals-4573954-supplementary.pdf]

# Supplementary Materials

## The Forgotten First Contact: The Oral Cavity as a Site of Local Biotransformation Shaping the Chemical Form of Polyphenolic Drugs and Dietary Compounds

Reference and section numbering follows the main manuscript. The status of the content of these tables is stated explicitly. Table S1 lists candidate determinants of the proposed oral metabotype; every entry is a hypothesis formulated by the authors, and none has been tested for polyphenolic substrates in the oral cavity. Table S2 assesses the suitability of experimental models and does not report results. Table S3 lists research priorities, not findings. The oral metabotype and the associated oral hydrolytic capacity classification are testable methodological proposals and not established biological phenotypes.

**Table S1.** Candidate determinants of the proposed oral metabotype, formulated as testable hypotheses; no determinant in this table has been shown to affect oral polyphenol conversion in humans.

| Determinant | Presumed mechanism | Modifiable |
|---|---|---|
| Periodontal status | alters the aerobic/anaerobic ratio and the enzymatic repertoire (Section 2.6.6) | yes |
| Salivary flow | determines contact time and the conversion achieved (Section 2.1.3) | partly |
| Oral hygiene and antiseptics | reduce biomass and, presumably, hydrolytic capacity | yes |
| Smoking | alters microbiota composition and oral redox state | yes |
| Plaque index and lingual biomass | determine total enzymatic mass | yes |
| Age | alters salivary flow and community composition | no |
| Habitual diet | enzymatic selection through repeated substrate exposure | yes |
| Recent antibiotic therapy | directly suppresses bacterial hydrolytic activity [8,9] | transiently |

**Table S2.** Suitability of the available models for the study of oral biotransformation of polyphenols. Reference numbers follow the main manuscript.

| Model | Represents the biofilm | Realistic time window | Host/microbiome discrimination | Applied to polyphenol biotransformation | Ref. |
|---|---|---|---|---|---|
| Whole saliva ex vivo | no | variable | through controls | yes | [8–10,35,38] |
| Microflora-reduced saliva | no | yes | yes | yes | [35] |
| Multispecies biofilm in vitro | yes | yes | partly | no; effects on the biofilm only | [54–56,89] |
| Anaerobic subgingival biofilm | yes | yes | partly | no | — |
| In vivo rinse with serial saliva | partly | yes | no | yes | [10,11,35] |
| Intraoral in situ device | yes | yes | no | no | — |
| Recombinant enzyme from an oral taxon | no | no | yes | partly (aroma, tannins) | [40–42] |
| Rapid test on the lingual surface | partly | yes | no | proof of principle | [9] |

**Table S3.** Prioritised research agenda. The objectives listed are proposed studies; the columns on feasibility and potential impact are the authors' estimates and not measured quantities.

| No. | Objective | Proposed design | Feasibility | Potential impact | Prediction tested |
|---|---|---|---|---|---|
| 1.1 | Population distribution of oral hydrolytic capacity | cohort n ≥ 150, LC-MS/MS kinetics | high | decisive for the proposed concept | (1) |
| 1.2 | Status of rutin as a substrate | standardised ex vivo, n ≥ 30 | high | moderate | — |
| 1.3 | Chlorhexidine interference | crossover, n = 20, seven days | high | high, translational | (3), (5) |
| 1.4 | Free fraction in saliva | ultrafiltration + LC-MS/MS | high | methodologically decisive | — |
| 2.1 | Enzyme–taxon attribution on polyphenolic substrates | metagenomics, metatranscriptomics, isolation | medium | high | — |
| 2.2 | Conversion in biofilm | multispecies model in vitro | high | high | — |
| 2.3 | Subgingival urolithins | anaerobic incubation and LC-MS/MS | medium | potentially the most informative | — |
| 2.4 | Oral compared with intestinal | dual phenotyping, equivalence test | medium | high | (4) |
| 3.1 | Stratification of trials | reanalysis and prospective design | low | high | (2) |
